# ELA-11 protects the heart against oxidative stress injury induced apoptosis through ERK/MAPK and PI3K/AKT signaling pathways

**DOI:** 10.3389/fphar.2022.873614

**Published:** 2022-09-08

**Authors:** Xuejun Wang, Li Zhang, Mengwen Feng, Zhongqing Xu, Zijie Cheng, Lingmei Qian

**Affiliations:** ^1^ Hongqiao International Institute of Medicine, Tongren Hospital, Shanghai Jiao Tong University School of Medicine, Shanghai, China; ^2^ Department of Cardiology, The First Affiliated Hospital of Nanjing Medical University, Nanjing, China

**Keywords:** ELA-11, doxorubicin, heart failure, apoptosis, oxidative stress

## Abstract

Increasing evidence revealed that apoptosis and oxidative stress injury were associated with the pathophysiology of doxorubicin (DOX)-induced myocardial injury. ELABELA (ELA) is a newly identified peptide with 32 amino acids, can reduce hypertension with exogenous infusion. However, the effect of 11-residue furn-cleaved fragment (ELA-11) is still unclear. We first administrated ELA-11 in DOX-injured mice and measured the cardiac function and investigated the effect of ELA-11 *in vivo*. We found that ELA-11 alleviated heart injury induced by DOX and inhibited cardiac tissues from apoptosis. *In vitro*, ELA-11 regulated the sensitivity towards apoptosis induced by oxidative stress with DOX treatment through PI3K/AKT and ERK/MAPK signaling pathway. Similarly, ELA-11 inhibited oxidative stress-induced apoptosis in cobalt chloride (CoCl_2_)-injured cardiomyocytes. Moreover, ELA-11 protected cardiomyocyte by interacting with Apelin receptor (APJ) by using 4-oxo-6-((pyrimidin-2-ylthio) methyl)-4H-pyran-3-yl 4-nitrobenzoate (ML221). Hence, our results indicated a protective role of ELA-11 in oxidative stress-induced apoptosis in DOX-induced myocardial injury.

## Introduction

Myocardial injury caused by chemotherapy drugs is a major reason that affecting the prognosis of tumor patients (Zhang et al., 2019). Statistically, 70% of patients in worldwide have different degrees of cardio-toxic reactions during chemotherapy, and these symptoms will accompany them for life (Leemasawat et al., 2020). In China, nearly 30% of cancer patients die from cardiovascular diseases. The most common cardio-toxicity caused by anthracycline is heart failure, with an incidence of 48% (Wu et al., 2017). It is known that the mechanism of doxorubicin (DOX)-induced cardiotoxicity is very complex, which involving pathological processes such as cell apoptosis, oxygen free radical damage, iron ion metabolism disorder, calcium overload and metabolic disorder (Kostrzewa-Nowak et al., 2005; Govender et al., 2014; Eid et al., 2021). Oxygen free radical injury is one of the representative theories of cardiotoxic injury caused by DOX (Ma et al., 2020). Cytochrome P450 reductase and a variety of reductases can produce superoxide free radicals (O2-) and reduce anthraquinone of DOX to form quinone-semiquinone cycle, leading to lipid peroxidation of mitochondria and microsomes, then damages myocardial cells (Yuan et al., 2020). Due to the decreased content of antioxidant enzymes in cardiomyocytes, a large number of reactive oxygen species and free radicals are generated to induce the oxidative stress response of cardiomyocytes and aggravate the damage of cardiomyocytes (Carrasco et al., 2021). Free radicals generated by DOX activated NAD(P)H oxidases [NAD(P)H oxidases, NOXs] can also activate the apoptosis pathway of cardiac myocytes and cause cell death (Wang et al., 2018). Furthermore, Pharmacological strategies that inhibit apoptosis and oxidative stress injury can protect patients from chemotherapeutic drug-induced myocardial damage.

Oxidative stress is an accompaniment of apoptosis through activating mitochondrial dysfunction, the death receptor pathway or endoplasmic reticulum stress (Dai et al., 2014). When cells are stimulated by oxidative stress, the accumulation of oxidizing substances such as free radicals can damage organelles and activate cell death program (Lüscher, 2015). Recent studies have demonstrated that the increase of intracellular ROS levels could put cells in a state of constitutive oxidative stress, leading to cell apoptosis (Lee et al., 2019). Targeting cell death pathways before oxidative stress manifestation can alleviate oxidative impairment by prevented free radicals which could potentially pave the way for new therapeutics through against oxidative stress induced apoptosis (Münzel et al., 2010; Malla et al., 2020).

It is well known that peptides produced by proteasomes degradation can play a protective role *in vivo* and act as endogenous ligands or receptors to mediate a variety of signaling pathways (Ye et al., 2019). For example, endorphins as an endogenous peptide which can ease pain, like morphine and analgesic (Paes et al., 2019). Angiotensin II binds to angiotensin receptors to regulate water-salt balance and blood pressure in heart and kidney (Johnson et al., 2017). Furthermore, natriuretic peptide family members can be used to diagnose heart failure (Brady et al., 2019). In conclusion, peptides play indispensable roles in regulating physiological function and pathophysiological process.

It has been reported that a conserved gene, AK092578, is predicted to encode a 54aa hormone with a signal peptide (Yang et al., 2017). This hormone is highly conserved among multiple species which is named ELABELA (ELA) or toddler (Read et al., 2019). As an early endogenous ligand of the Apelin receptor (APJ), ELA is essential for heart development. In recent studies, it has been suggested that a 32-amino-acid mature peptide (ELA-32) can be decomposed to produce endogenous fragments including ELA-21 and ELA-11 (Chen et al., 2017). Studies have proved that ELA-32 and ELA-11 could inhibit renal ischemia-reperfusion (I/R) injury, and ELA-21 could significantly increase angiogenesis, promoted cardiomyocyte proliferation and reduced apoptosis and heart fibrosis near the infarct area (Xu, 2021). Recent study has clarified that Elabela (19–32) could ameliorate doxorubicin (DOX)-induced cardiotoxicity by promoting autophagic flux through TFEB pathway (Chen et al., 2022). However, the effect and mechanism of ELA-11 in DOX-induced cardiac injury is unclear.

The PI3K-AKT signaling pathway is a classical signaling pathway that regulates apoptosis (Brady et al., 2019). PI (3, 4, 5) P_3_ is an intracellular second messenger in the cell that is required for the transfer of protein kinase B (AKT) to the membrane for activation (Lin et al., 2019). Phosphorylation of AKT mediates insulin and various growth factors to induce cell growth and promotes cell survival through numerous channels (Song et al., 2018). ELA-11 induced ERK/MAPK is a classical anti-apoptotic signaling pathway. When the downstream phosphorylation of ERK is activated, it inhibits the process of apoptosis.

In present study, we found that ELA-11 could attenuate DOX-induced free radical production, which protected cardiomyocytes against oxidative stress-induced apoptosis. Mechanistically, ELA-11 inhibited oxidative stress-induced apoptosis by suppressing mitochondrial membrane potential mediated by ERK/MAPK and PI3K/AKT signaling pathways. Moreover, ELA-11 acted as a protective role in DOX-induced cardiac injury by targeting APJ.

## Materials and methods

### Cell culture

Rat primary cardiomyocytes were extracted from rats 24 h after birth. The blood, fat, connective tissues and heart tissue sections were separated and digested with trypsin at 37°C at 60 rpm for 15 min. The solution was removed, and the digestion was repeated three times. The cell-containing digested fluids were placed in a centrifuge tube and centrifuged together after passing through a 180-mesh filters. The cells were resuspended in 10 ml DMEM containing 10% horse serum (HS, Gibco, United States) and incubated in a 10 cm^2^ dish for 1.5 h. The cell suspension was removed, and the cells (5–6 × 10^5^ cells per dish) were inoculated into a new dish as previously described. When cell adhered to about 80%, 1uM ELA-11 was added to the culture media. After 12 h of co-incubation with DOX, experimental verification was carried out.

### Animals

Male C57BL/6J mice (6–8 weeks of age, 20–22 g) were obtained from the Model Animal Research Center of Nanjing University (Nanjing, Jiangsu, China), and all procedures used were approved by the ethical committee of Nanjing Medical University. All animals were housed at 20–25°C and 50%–70% relative humidity. The experimental mice were randomly divided into four groups. Before injected with 5 mg/kg DOX by intraperitoneal injection for five consecutive weeks, 10 mg/kg ELA-11 was injected to mice through veil tail for 7 days, after which electrocardiograms were obtained and the mice were sacrificed. The mice were treated according to the experimental requirements. All animal experiments complied with the Guide for the Care and Use of Laboratory Animals published by the National Institutes of Health (NIH Publications No. 85-23, revised 1996).

### Cell-in-cell experiment

We chemically synthesized FITC-labeled, incubated cells with ELA-11 for 1 h at 37°C and 5% CO_2_ atmosphere. The localization of ELA-11 was observed by fluorescence microscopy.

### Cell counting kit-8 assay

Thousand cells were inoculated into each well of the 96-well plate, ELA-11, DOX or CoCl_2_ was added into the plates respectively after cells were adhered. Measured the absorbance at 450OD. Cell viability (%) = [OD (experimental group)–OD (blank group)]/[OD (control group)–OD (blank group)] * 100%.

### Cell death rates

Trypan blue staining was used to calculate the mortality of the primary cells. The cells were collected at different times (0, 6, 12, 18, 24 or 36 h) and stained with the dye from a trypan blue staining cell viability assay kit to determine the cell death rate. At different concentrations of DOX (0.1, 0.5, 1, 2, and 5 µM) and CoCl_2_ (200, 400, 600, 800 and 1,000 µM), measurements were taken according to the manufacturer’s instructions.

### Lactate dehydrogenase level detection

Levels of lactate dehydrogenase (LDH) released were detected in serum using an LDH release assay kit according to the manufacturer’s protocol.

### Terminal-deoxynucleotidyl transferase-mediated dUTP-biotin nick end-labeling staining assay

The rate of apoptosis can be was detected by a TUNEL staining kit. The cells were seeded (1 × 10^5^ cells per well) in 6-well dishes. After the treatments described above were performed, the cells were washed once with phosphate-buffered saline (PBS) and fixed with 4% paraformaldehyde. Apoptotic cells were visualized with TUNEL staining according to the manufacturer’s protocol. TUNEL fluorescence intensity/DAPI fluorescence density was used to calculate the percentage of positive cells, and the density was evaluated using ImageJ software 1.26 (Wayne Rasband, National Institutes of Health, Bethesda, MD, United States).

### Tetrechloro-tetraethylbenzimidazol carbocyanine iodide assay

The mitochondrial membrane potential was measured by a mitochondrial membrane potential assay kit with JC-1 according to the manufacturer’s instructions. The cells were cultured in serum-free DMEM containing (×1) JC-1 staining working fluid at 37°C for 20 min. Then, the cells were washed twice with JC-1 buffer, after which 2 ml DMEM was added, and then the cells were photographed with a fluorescence microscope (BX61; Olympus Corporation, Tokyo, Japan). The JC-1 density was assessed by ImageJ software and calculated upon normalization to the control.

### Reactive oxygen species measurement

The levels of intracellular ROS were determined using a relative oxygen species assay kit following the instructions. Cells were incubated in serum-free DMEM containing 0.1% DCFH-DA at 37°C for 20 min, washed with serum-free DMEM three times and photographed with a fluorescence microscope.

### Western blot

Proteins were isolated from cells using lysis buffer (containing RIPA and 1% PMSF). Protein quantification was performed using a BCA protein detection kit (23229; Thermo Fisher Scientific). Protein samples of the same mass were separated on 10% SDS-PAGE gels and transferred to nitrocellulose membranes (Millipore, Billerica, MA, United States), which were blocked with 5% skim milk and then incubated with specific primary antibodies. Caspase-3 (14220T, 1:1000), PARP (9532T, 1:1000), β-actin (3700S, 1:1000), GAPDH (5174T, 1:1000), AKT (4685S, 1:1000), p-AKT (4060T, 1:2000), PI3K (4249T, 1:1000), ERK (4695T, 1:1000) and p-ERK (4370T, 1:1000) were purchased from Cell signaling Technology. The anti-rabbit (SA00001-2, 1:10000), PI3K (20584-1-AP, 1:1000) and anti-mouse (SA00001-1, 1:10000) secondary antibody were purchase from Proteintech. FluorChem M system was used to quantify the positive bands representing proteins involved in the orchestrated immune responses (ProteinSimple, San Jose, CA, United States).

### Malondialdehyde, superoxide dismutase, and glutathione peroxidase assay

Cellular oxidative stress was determined by detecting the state of intracellular oxidation and reduction. Lipid peroxidation Malondialdehyde (MDA) assay (Beyotime, Nanjing, China) and total superoxide dismutase assay (Beyotime, Nanjing, China) were measured by following the manufacturer’s instructions. glutathione peroxidase (GSH-Px) assay kit was purchased from Elabscience. The cell supernatant was collected, and the standard substance was prepared and diluted according to the instructions, the concentration of each well was 600, 400, 200, 100, and 50 U/L, respectively. The tested samples were added to the plate and mixed, then incubated at 37°C for 30 min. Wash plates, add enzyme and chromogenic reaction were proceeded according to the instructions. After incubating at 37°C for 15 min, the absorbance of each well was measured at 450 nm wavelength.

### Echocardiography

Post DOX and ELA-11 administration, all the mice were subjected to M-mode echocardiography to assess heart function. All animal experiments complied with the Guide for the Care and Use of Laboratory Animals published by the National Institutes of Health (NIH Publications No. 85-23, revised 1996).

### Enzyme-linked immunosorbent assay

Serum Creatine kinase isoenzyme (CKMB) and B-type natriuretic peptide (BNP) level in mice heart tissue was determined by a commercially available Enzyme-linked immunosorbent assay (ELISA) kit (Mlbio, Shanghai, China) according to the manufacturer’s instructions.

### Hematoxylin & Eosin (H&E) Staining

Obtained the cardiac tissues and fixed them in 4% paraformaldehyde for 7 days after the blood in the heart cavity was pumped out. The tissue was transparent with ethanol and xylene. Soak the transparent tissue into melted paraffin for embedding. After cooling and solidification, they were cut into five micron slices and placed in hot water to flatten and then pasted onto slides and dried in a 45°C incubator. Before staining, paraffin was removed again, and Hematoxylin and eosin dyes were respectively used for staining, which made the nucleus and intracellular ribosomes stained blue and purple by Hematoxylin (H). The cytoplasm is stained red or reddish with Eosin (E).

### Masson staining

Obtained and sectioned tissue according to the above method. Weigert sapwood semen was used for staining nuclear for 5–10 min, and ponceau acid fuchsin solution was used for 5–10 min after washing. Soaked with 2% glacial acetic acid solution for 30 s, and differentiate with 1% phosphomolybdate solution for 3–5 min. Then, the slices were dyed with aniline blue for 5 min, and bathed again with 0.2% glacial acetic acid solution for 30 s. Finally, the slices were sealed with 95% alcohol, anhydrous alcohol, xylene transparent and neutral gum.

### Wheat germ agglutinin staining

Obtained and sectioned tissue according to the above method. Placed the section in the antigen repair buffer of pH 8.0, then the section was decolorized with PBS after cooled naturally. A histochemical pen was used to draw circles around the tissues and Wheat germ agglutinin (WGA) staining buffer was added into the circles, and the cells were incubated for 30 min at 37°C. The nucleus was stained with DAPI. Sealed the section, then collected the images under fluorescence microscope.

### Statistical analysis

All results are expressed as the mean ± SD. Comparisons between multiple groups were performed by one-way analysis of variance (ANOVA), and *p* < 0.05 (*), *p* < 0.01 (**) and *p* < 0.001 (***) were considered significant. All experiments were repeated at least three times unless otherwise specified. All data was analyzed by GraphPad Prism software.

## Results

### Function of ELA-11 in doxorubicin-injured model

**FIGURE 1:**
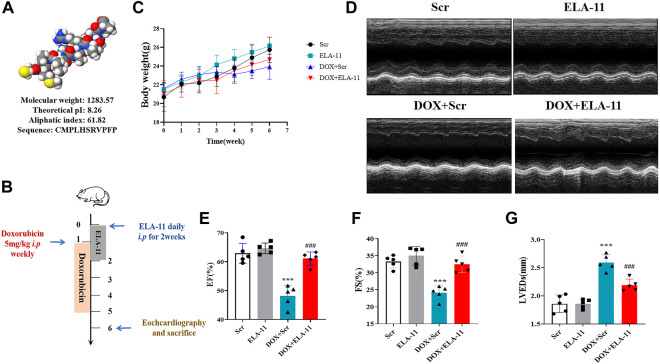
Function of ELA-11 in doxorubicin-injured model. **(A)** The biological characteristics of ELA. **(B)** Schematic map for DOX-induced mice cardiac injury model. **(C)** The body weight of mice in four groups during the experiment. **(D)** The representative echocardiograms of DOX-induced cardiac injured group. **(E)** EF value for echocardiography. **(F)** FS value for echocardiography. **(G)** LVEDs value for echocardiography. ***p < 0.001 and ^###^ *p* < 0.001, one-way ANOVA, N = 5 mice per group.

To understand the shape of ELA-11 in molecular chain, we predicted its structure by I-TASSER server (https://zhanggroup.org/I-TASSER/) and analyzed the biological characteristics through Expasy (https://www.expasy.org/) (Figure 1A). We found that ELA-11 possessed the advantages of light molecular weight and high lipid solubility. Hence, we investigated the effect of ELA-11 in DOX-induced cardiac injury *in vivo* (Figure 1B). We weighed the mice every 2 weeks for 10 weeks, the result demonstrated that the weight of DOX-induced group began to decrease after 6 weeks, scramble (scr) peptide could not affect cell death, but DOX increased cell death since week 5. Compared with DOX, ELA-11 could increase weight index significantly (Figure 1C). Next, the echocardiographic results suggested that DOX reduced ejection fraction (EF) and fractional shortening (FS), but increased left ventricular end-systolic dimension (LVED) significantly, however, ELA-11 increased EF and FS, and decreased left ventricular end-systolic dimension (LVED) significantly compared with DOX + Scr group (Figures 1D–G). These results demonstrated that ELA-11 could inhibit DOX-induced injury *in vivo*.

### ELA-11 protects doxorubicin-injured heart in vivo

**FIGURE 2:**
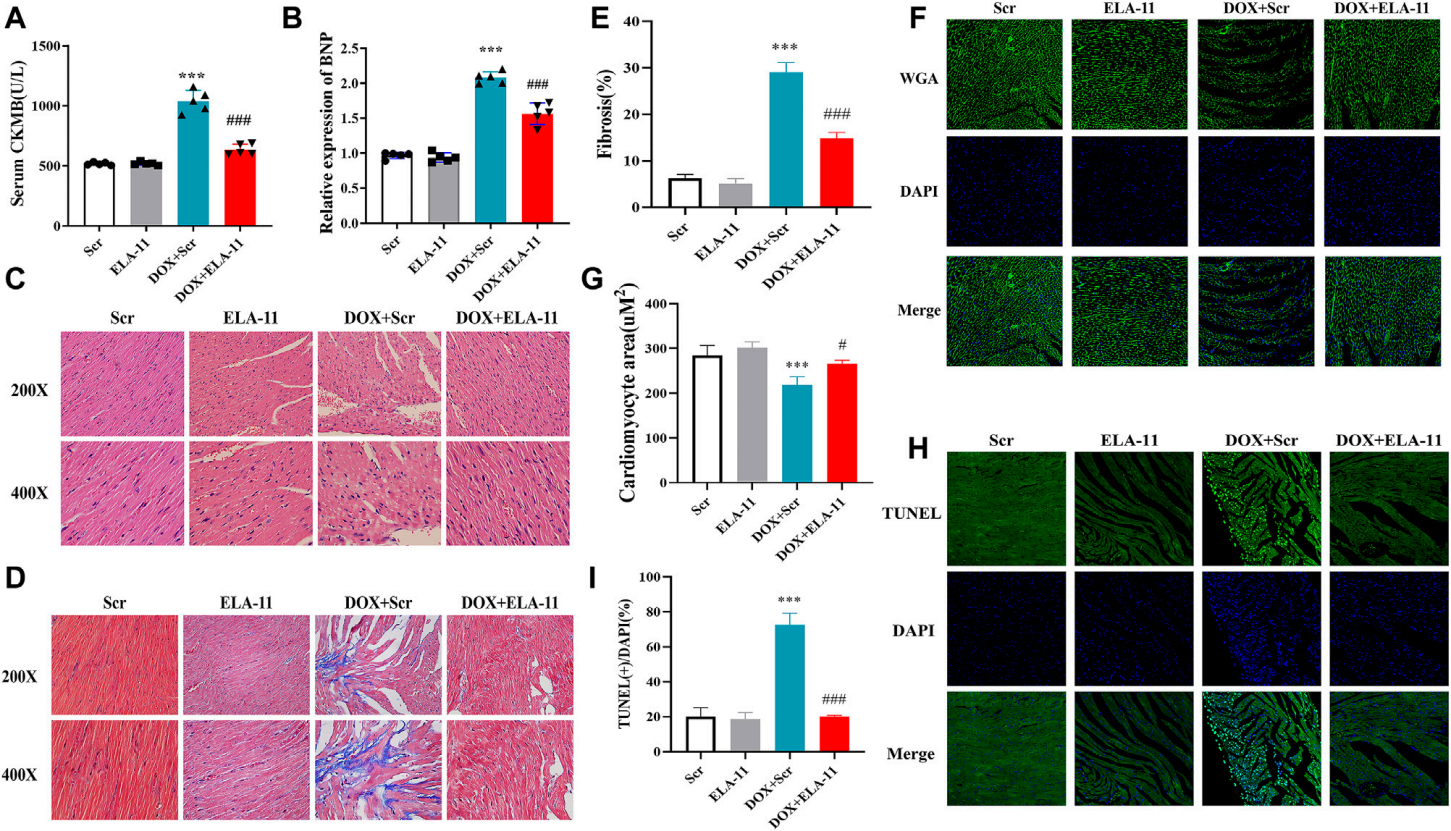
ELA-11 protects doxorubicin-injured heart in vivo. **(A)** Serum CKMB release in DOX-injured model. **(B)** Serum BNP release in DOX-injured model. **(C)** Representative photographs of HE staining. **(D)** Representative photographs of Masson staining. **(E,F)** Representative photographs of WGA staining and its quantitative data. **(G,H)** Representative photographs of TUNEL staining and its quantitative data. ***p < 0.001 and ^###^ *p* < 0.001, one-way ANOVA, N = 5 mice per group.

Then, our results demonstrated that the release of CKMB and BNP in blood serum increased significantly in DOX + Scr treated group compared with Scr group, but ELA-11 decreased CKMB and BNP release significantly (Figures 2A,B). To further identify the effect of ELA-11 *in vivo*, we collected organs to perform pathological examination. HE staining results indicated that myocardial fibers in DOX-treated group were in disordered arrangement and cardiomyocytes were changed into vacuolated compared with Scr group, but there was less disarrayed myocardial fibers and vacuolated cells in DOX + ELA-11 treated group (Figure 2C). The evidence from Masson staining suggested that fibrosis was significantly increased by DOX but reduced by ELA-11 (Figures 2D,E). To figure out the cardiomyocyte areas of the four groups, WGA staining results demonstrated that the cardiomyocyte area decreased significantly in DOX-treated group compared with the Scr group, but ELA-11 significantly rescued cardiomyocyte area (Figures 2F,G). TUNEL staining showed a significant increase in the apoptosis rate in the myocardium in the DOX + Scr group, and it generally decreased to the level in DOX mice administered with ELA-11 (Figures 2H,I). These results demonstrated that ELA-11 might have a protective role in DOX-induced cardiac injury.

### ELA-11 attenuates doxorubicin-induced injury in rat primary cardiomyocytes

**FIGURE 3:**
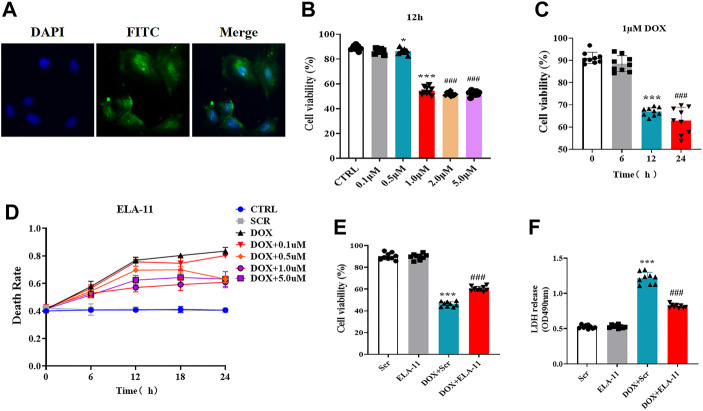
ELA-11 attenuates doxorubicin-induced injury in rat primary cardiomyocytes. **(A)** The location of ELA-11 in rat primary cardiomyocytes. **(B)** Trypan blue assay for cell death with different concentration of DOX with different duration (N = 9 per group). **(C)** The cell viability of 1 uM DOX in different duration (N = 9 per group). **(D)** Cell viability of ELA-11 with different concentration for 12 h (N = 9 per group). **(E)** Cell viability in DOX-induced cardiomyocyte model (N = 9 per group). **(F)** LDH release in DOX-induced cardiomyocyte model (N = 9 per group). ***p < 0.001 and ^###^ *p* < 10.001, one-way ANOVA.

To determine the effect of ELA-11 *in vitro*, we first cultured rat primary cardiomyocytes with ELA-11, we found that ELA-11 could enter into the cytoplasm (Figure 3A). To figure out the optimal concentration of DOX, we incubated DOX with different concentrations (0.1, 0.5, 1, 2, and 5 uM) for 12 h, CCK-8 results demonstrated that 0.5 uM DOX could reduce cell viability, but 1, 2, and 5 uM decreased cell viability more significantly and there were no difference among 1, 2 and 5 uM DOX (Figure 3B). Then CCK-8 results manifested that 1 uM DOX cultured for 12 and 24 h could effectively reduce the cell survival rate, however there was no statistic difference between the two groups (Figure 3C). To figure out whether ELA-11 could affect cell survival, we cultured ELA-11 with different duration times (0, 6, 12, and 24 h) and concentrations (0.1, 0.5, 1, 2, and 5 uM). Then trypan blue dyeing results demonstrated that 0.5 uM ELA-11 decreased cell death at 24 h, 1 uM, and 5 uM ELA-11 both could decrease cell death at 12 h. But there was no significant difference among the groups (Figure 3D). Next, we cultured 1 uM ELA-11 before DOX treatment for 12 h and found ELA-11 could significantly increase cell viability compared with DOX + Scr group (Figure 3E). And LDH results indicated that ELA-11 inhibited DOX-induced cardiotoxicity (Figure 3F). Our study revealed that ELA-11 could attenuate DOX-induced injury in rat primary cardiomyocytes.

### ELA-11 inhibits doxorubicin-induced cardiac injury through oxidative stress-induced apoptosis

**FIGURE 4:**
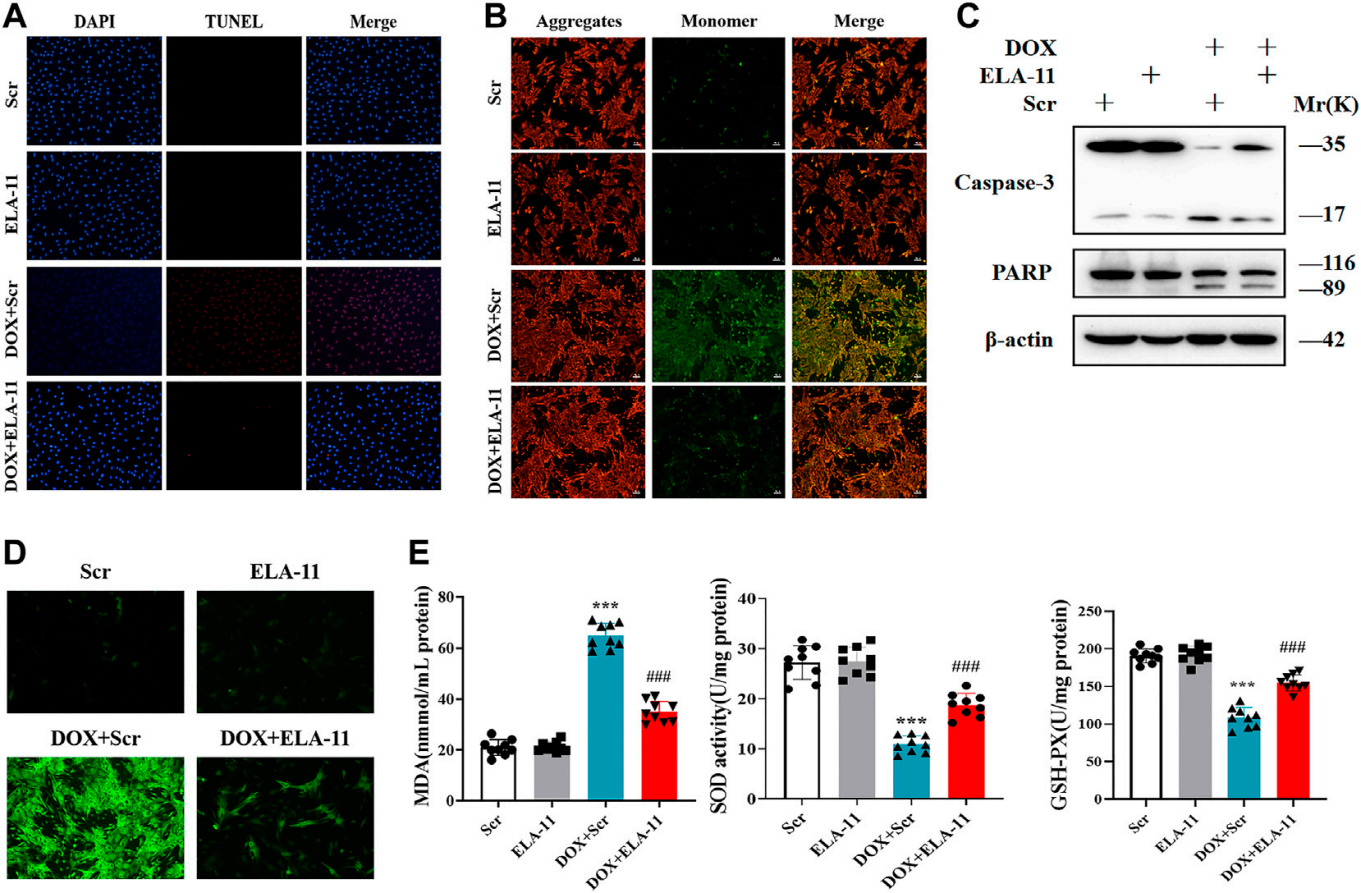
ELA-11 inhibits DOX-induced cardiac injury through oxidative stress -induced apoptosis. **(A)** Representative photographs of TUNEL staining in DOX- induced cardiomyocyte injury (N = 9 per group). **(B)** Representative photographs of JC-1 in DOX-induced cardiomyocyte injury. **(C)** The expression of PARP and caspase-3 in DOX-induced cardio-myocyte injury by western blot. **(D)** Representative photographs of ROS in DOX-induced cardio-myocyte injury. **(E)** Detection of MDA production, SOD activity and GSH-PX release in DOX-induced cardiomyocyte injury (N = 9 per group). ***p < 0.001 and ^###^ *p* < 0.001, one-way ANOVA.

Based on the effect of ELA-11 *in vivo*, we further detected the apoptosis level by TUNEL, the results revealed that the number of apoptotic cells was increased in the DOX-induced group but reduced after ELA-11 co-treatment (Figure 4A). Furthermore, DOX reduced mitochondrial membrane potential compared with scr group, but ELA-11 elevated mitochondrial membrane potential compared with DOX treatment (Figure 4B). The western blot results revealed that cleaved caspase-3 and PARP were activated by DOX, but ELA-11 reduced the elevated expression of cleaved caspase-3 and PARP (Figure 4C). Next, ROS release results indicated that ELA-11 attenuated DOX-induced free radical production (Figure 4D). Furthermore, we evaluated cellular oxidative stress levels by MDA production, SOD activity and GSH-Px content. Our results demonstrated that DOX increased MDA production and reduced SOD activity and GSH-Pxproduction, but ELA-11 inhibited MDA production, promoted SOD activity and GSH-Px production (Figure 4E). Therefore, ELA-11 could inhibit DOX-induced injury by attenuating oxidative stress-induced apoptosis.

### ELA-11 protected cardiomyocytes against oxidative stress-induced apoptosis in CoCl_2_ model

**FIGURE 5:**
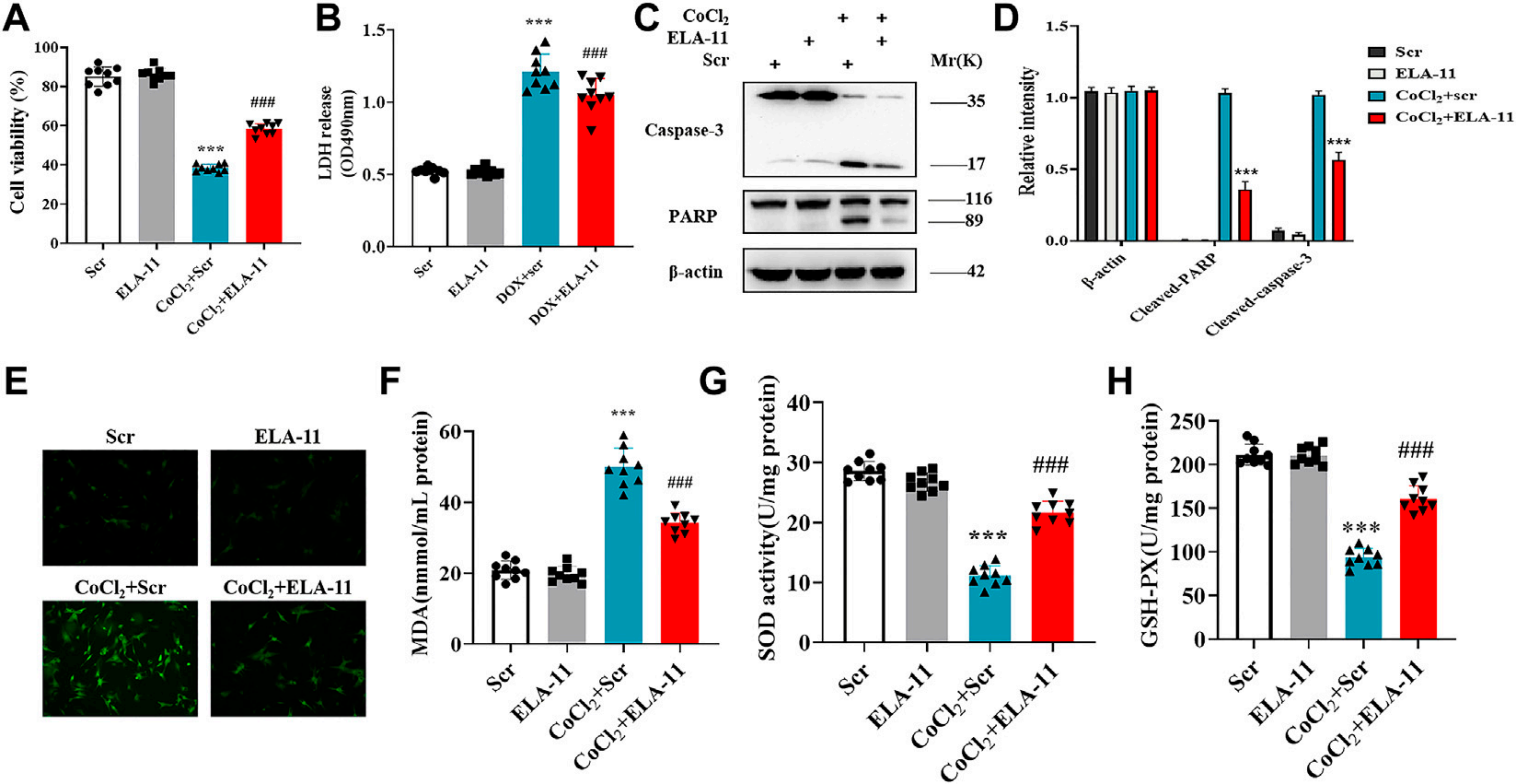
ELA-11 protected cardiomyocytes against oxidative stress-induced apoptosis in CoCl_2_ model. **(A)** Cell viability in CoCl_2_ -induced cardiomyocyte model. **(B)** LDH release in CoCl_2_ -induced cardiomyocyte oxidative stress injury (N = 9 per group). **(C,D)** The expression of PARP and caspase-3 in CoCl_2_ -induced cardiomyocyte oxidative stress injury by western blot. **(E)** Representative photographs of ROS production in CoCl_2_ -induced cardiomyocyte oxidative stress injury. **(F–H)** Detection of MDA production, SOD activity and GSH -PX release in CoCl_2_ -induced cardiomyocyte oxidative stress injury (N = 9 per group). ***p < 0.001 and ^###^ *p* < 0.001, one-way ANOVA.

To validate the role of ELA-11 in another oxidative stress model, we further constructed chemical ischemic model in rat primary cardiomyocytes. Our results indicated that ELA-11 protected cardiomyocytes from CoCl_2_-induced injury (Figures 5A,B). Then western blot evidence proved that ELA-11 could inhibit CoCl_2_-induced cardiac apoptosis (Figures 5C,D). Furthermore, ELA-11 inhibited ROS production with CoCl_2_ treatment (Figure 5E). And ELA-11 inhibited superoxide production and peroxidase activity (Figures 5F–H). Our results further proved ELA-11 protected cardiac injury in CoCl_2_ model mediated by oxidative stress-induced apoptosis.

### ELA-11 attenuated cardiac injury through ERK/MAPK and PI3K/AKT signaling pathway

**FIGURE 6:**
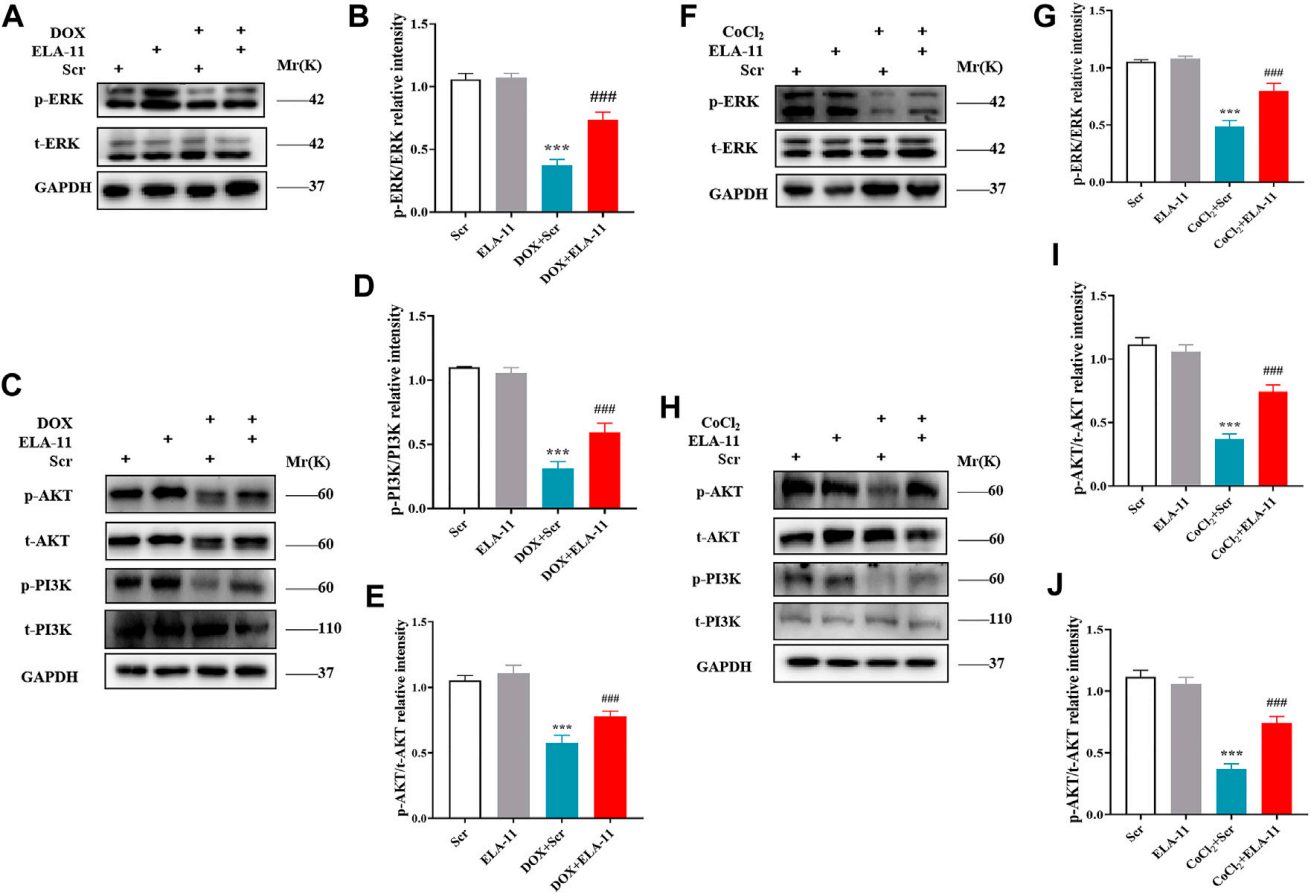
ELA-11 attenuated cardiac injury through ERK/MAPK and PI3K/AKT signaling pathway. **(A)** The expression of ERK in DOX-induced cardiac injury by western blot. **(B)** The quantitative data for western blot. **(C–E)** The expression of PI3K and AKT in DOX-induced cardiac injury by western blot and its quantitative data. **(F,G)** Western blot analyzed the expression the expression of ERK in CoCl2 -induced cardiac injury and its quantitative data. **(H–J)** Western blot analyzed the expression the expression of PI3K and AKT in CoCl_2_ -induced cardiac injury and its quantitative data. ***p < 0.001 and ^###^ *p* < 0.001, one-way ANOVA.

We further verified the possible signaling pathway of ELA-11 in rat primary cardiomyocytes by Western blot. We first determined the expression of p38, JNK and ERK, however, the result showed that there was no expression of JNK and p38 (data not shown), but phosphorylated ERK was down-regulated by DOX and ERK protein phosphorylation was activated upon ELA-11 treatment compared with DOX (Figures 6A,B). Next, we found that DOX also down-regulated the expression of phosphorylated AKT and PI3K, but ELA-11 activated phosphorylation of AKT and PI3K protein expression (Figures 6C–E). When CoCl_2_ was blunted, the phosphorylation of ERK, AKT and PI3K protein expression was downregulated. With intervention of ELA-11, ELA-11 activated the expression of ERK, AKT and PI3K phosphorylated proteins after CoCl_2_ treatment in rat primary cardiomyocytes (Figures 6F–J). These results revealed that ELA-11 protected cardiomyocytes from apoptosis through the ERK/MAPK and PI3K/AKT signaling pathways.

### ELA-11 protects cardiomyocytes by binding apelin receptor

**FIGURE 7:**
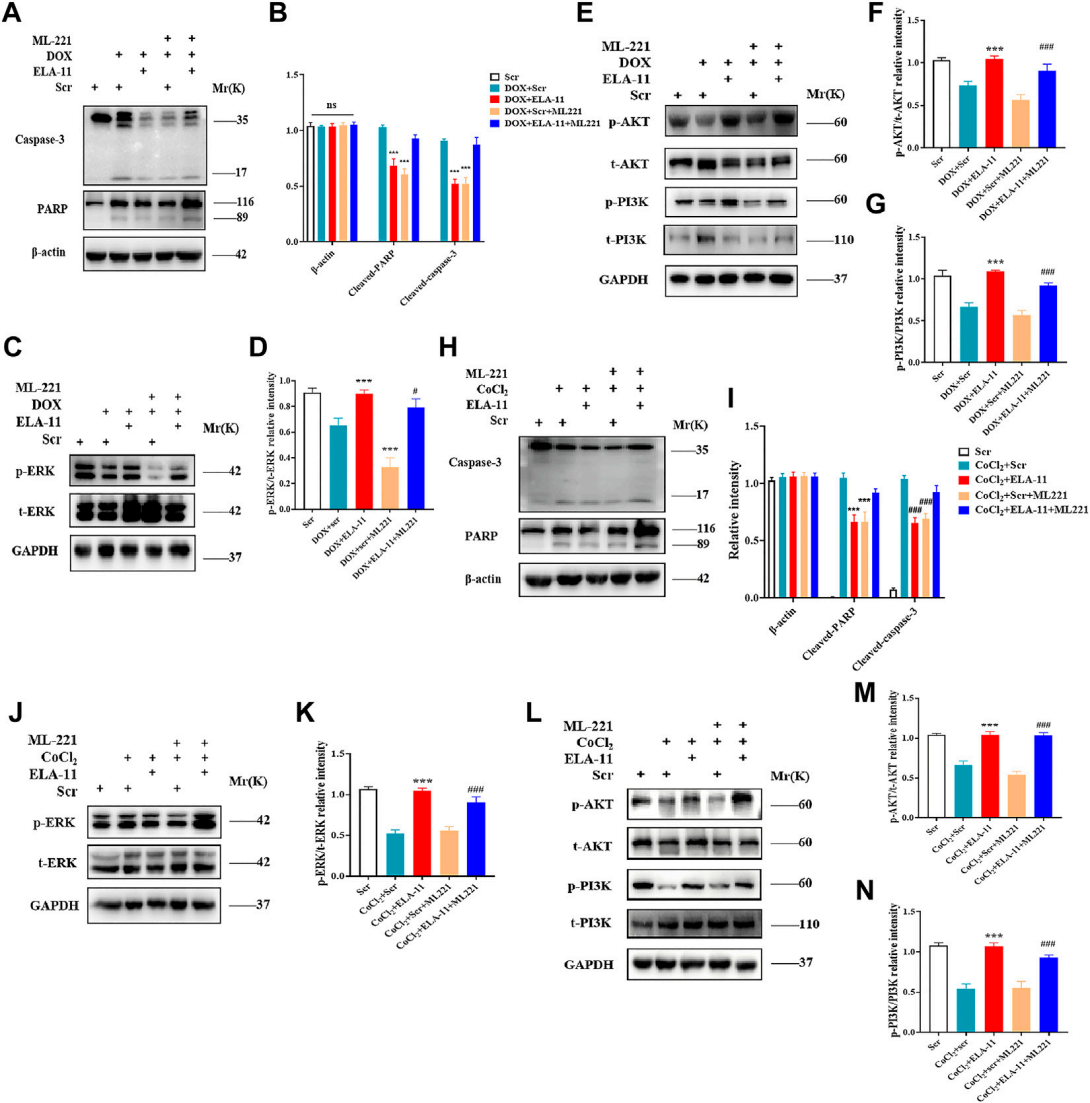
ELA-11 protects cardiomyocytes by binding APJ. **(A,B)** Western blot result of PARP and caspase-3 with ML221 treatment in DOX-induced group and its quantiied data. **(C,D)** Western blot result of ERK with ML221 treatment in DOX -induced group and its quantiied data. **(E–G)** Western blot and quantiied data for PI3K and AKT in DOX-induced group. **(H,I)** The expression of PARP and casapse-3 in CoCl_2_ -induced group by western blot and quantiied. **(J,K)** The expression of ERK in CoCl_2_ -induced group by western blot and quantiied. **(L–N)** The expression of PI3K and AKT in CoCl_2_ -induced group with quantiied data. ***p < 0.001, and ^###^ *p* < 0.001, one-way ANOVA.

**FIGURE 8:**
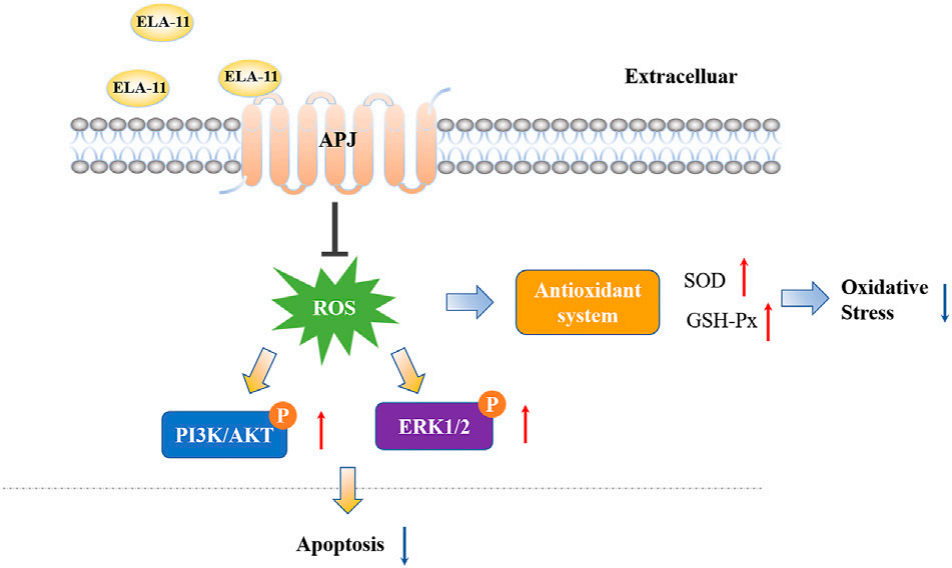
ELA-11 platform for inhibiting apoptosis and oxidative stress injury in cardiomyocytes.

4- oxo-6-((pyrimidin-2-ylthio) methyl)-4H-pyran-3-yl4-nitrobenzoate (ML221) is the first reported APJ antagonist which exerts antagonistic effect mainly by inhibiting cAMP and recruiting β-arrestin by ELA-11. Report has shown that ML221 has potential to attenuate the activation and signaling of the APJ receptor and reduce elabela-induced microvascular endothelial cell proliferation (Godoy-Parejo et al., 2019). To determine the role of ML221, we used to detect its interaction with ELA-11 by Western blot. We found that ML221 inhibited the effect of ELA-11 on cardiomyocyte apoptosis after DOX administration (Figures 7A,B). We wondered whether ML221 was involved in the ERK/MAPK and PI3K/AKT signaling pathways when the cells were treated with DOX, and western blot results demonstrated that ML221 significantly decreased the phosphorylation of AKT, PI3K, and ERK proteins compared with ELA-11, when ELA-11 and ML221 co-incubation with DOX, the ability of ELA-11 to activate phosphorylated ERK, PI3K and AKT was inhibited (Figures 7C–G). Furthermore, we found that ML221 could also inhibit the effect of ELA-11 in CoCl_2_-induced apoptosi s (Figures 7H,I). The phosphorylation of AKT, PI3K and ERK proteins was significantly up-regulated after co-treatment with ML221 and ELA-11 in CoCl_2_ model (Figures 7J,K,L–N). These results suggested that ELA-11 inhibited apoptosis by binding to APJ. Finally, we drew a pattern diagram of ELA-11 for inhibiting apoptosis and oxidative stress injury in cardiomyocytes (Figures 8).

## Discussion

Our current study revealed that ELA-11 has a protective effect on apoptosis and attenuates DOX-induced cardiotoxicity *in vitro* and *in vivo*. Furthermore, we demonstrated that ELA-11 attenuated oxidative stress-induced apoptosis through ERK/MAPK and PI3K/AKT signaling pathways by targeting APJ. ERK/MAPK and PI3K/AKT signaling pathways is regarded as a promising molecular mechanism in apoptosis, proliferation and differentiation. Our findings provide evidence of the molecular mechanism by which the ELA-11 peptide inhibits oxidative stress-induced apoptosis, indicating its potential use for DOX-induced cardiotoxicity.

Many known bioactive substances secreted by heart, including ANP, have been indicated to be involved in the regulation of cardiovascular disease and sensitive as indicators of disease monitoring (Ishimaru et al., 2017). In addition, endocrine-derived peptides play a dispensable role in different pathological processes and engage in organ crosstalk. For instance, a vasoactive intestinal polypeptide of 28 amino acids is released by intestinal neurons (Reginauld et al., 2019). Its level of variation is related to a variety of human diseases. It is worth observing that some long peptides can be cleaved into smaller peptides that have more consequential functions than those of longer peptides.

Because of the cardiac toxicity caused by DOX, its clinical application is severely limited (Sun et al., 2019). Apoptosis and oxidative stress injury induced by DOX came into our insight. CoCl_2_ is a classic and effective compound used to simulate hypoxic and ischemic processes (Ma et al., 2020). In consistence with previous studies, our study has demonstrated that DOX and CoCl_2_ can induce apoptosis and oxidative stress injury. APJ receptor is an essential orphan G protein-coupled receptor super-family. Previous studies have demonstrated that ELA-21 and ELA-32 involved in heart development and stem cell maintenance (Dai et al., 2008). ELA-11 is the shortest peptide in ELABELA family and may retain the functional peptide to perform biological functions (Ma et al., 2021). We proved that ELA-11 can also protect cardiac function by interacting with APJ.

As an endogenous ligand of Apelin, APJ plays a cardiovascular protective role by binding to different G protein subtypes and trigger multiple signaling pathways, such as AMPK, PI3K/Akt or MAPK signaling pathways (Li et al., 2021). Elabela was first identified as a novel ligand of APJ receptor in zebrafish embryos. The Ela-APJ axis is critical to a variety of biological processes and has been shown to regulate humoral homeostasis, myocardial contractility, vasodilation, angiogenesis, cell differentiation, apoptosis, oxidative stress, cardiorenal fibrosis and dysfunction (Sato et al., 2017). In human embryonic stem cells, Elabela can activate PI3K/Akt/MTORC1 signaling pathway to regulate self-renewal and survival of stem cells (Ho et al., 2015). As we all know that DOX induced cardiotoxicity involves a variety of molecular mechanisms, including energy metabolism, oxidative stress, and programmed cell death (Tocchetti et al., 2014). The excessed ROS can be effectively eliminated by the antioxidant defense system in physiological status (Zhang et al., 2022). However, when the antioxidant defense system is unable to consume excessive levels of ROS, cytotoxic signaling pathways will activate, leading to DNA damage, mitochondrial dysfunction and abnormal cellular calcium homeostasis.

Oxidative stress irritates the activity of ion exchangers, including Na+/H+ exchangers (NHE-1) and transient receptor potentials melastain 2 (TRPM2), resulting in overload of cell serum and mitochondrial Ca2+. Overloaded Ca2+ leads to activation of mitochondrial Ca2+ sensitive dehydrogenase and massive production of respiratory metabolic chain substrate NADH, which again promotes ROS production (Javadov et al., 2008). This cycle eventually leads to dyshomeostasis, apoptosis, inflammatory responses and other pathological process. Hence, ROS overproduction is the main event of oxidative stress injury (Schieber and Chandel, 2014). Mechanically, as an essential signaling pathway of G-protein-coupled receptors, APJ can activates RAF-1 to phosphorylate threonine and tyrosine through PI3K/AKT, and ultimately activate ERK1 and ERK2 (i.e., p44MAPK and p42MAPK) (Chiu et al., 2013). Therefore, AKT and ERK signaling pathways play a key role in oxidative stress response.

Multiple evidence indicated that mitochondrial permeability conversion pore (mPTP) plays a key role in regulating cardiac apoptosis under pathological conditions (Hou et al., 2022). Changes in intracellular pH, mitochondrial membrane potential or reactive oxygen species can regulate THE opening of mPTP (Ahmad et al., 2019). Glycogen synthase kinase (GSK)-3α/β is a serine/threonine kinase that regulates the opening of mPTP and participates in the apoptosis process of cardiomyocytes (Ahmad et al., 2014). The activation of PI3K/AKT signaling pathway may regulate the activation of GSK-3 downstream protein. Knock down the expression of GSK-3 in adult cardiomyocyte can lead to mitotic catastrophe which resulting in fatal dilated cardiomyopathy (Zhou et al., 2016; Ahmad and Woodgett, 2020). And inhibition of GSK-3αmay be a novel strategy for limited adverse ventricular remodeling and dysfunction after myocardial infarction in future treatment (Lal et al., 2015). Whether GSK-3 can play a role in DOX induced cardiotoxicity will be further explored in future studies.

Although recent studies have shown that the short peptide ELABELA (19–32) can ameliorate DOX-induced cardiotoxicity by promoting autophagic flux through TFEB pathway, ELA-11, shorter than ELABELA (19–32) reduced ROS release, thereby inhibiting mitochondrial oxidative stress and further inhibiting apoptosis of cardiomyocytes. Furthermore, In our study, the results supported that ELA-11 resisted cardiotoxicity by inhibiting apoptosis and oxidative stress by activating PI3K, AKT and ERK phosphorylation.

Our study also has some limitations. First, more clinical samples are needed to determine the exact window of time of interactions for clinical application. Second, whether the modification of ELA-11 influences its function in cardiovascular diseases needs further evaluation.

## Data Availability

The original contributions presented in the study are included in the article/Supplementary Materials, further inquiries can be directed to the corresponding authors.

## Abbreviations

LDH, lactate dehydrogenase; ROS, relative oxygen species; TUNEL assay, terminal-deoxynucleotidyl transferase (TdT)-mediated dUTP-biotin nick end-labeling assay; ELISA, enzyme-linked immunosorbent assay; DOX, doxorubicin; CoCl_2_, cobalt chloride; MDA, malondialdehyde; SOD, superoxide dismutase; ERK, extracellular signal-related kinase; JNK, Jun amino-terminal kinase; PI3K, phosphatidylinositol 3 kinase; AKT, protein kinase B; JC-1, tetrechloro-tetraethylbenzimidazol carbocyanine iodide; ML221, 4-oxo-6-((pyrimidin-2-ylthio) methyl)-4H-pyran-3-yl 4-nitrobenzoate; Scr, scramble; AA, amino acid; EF, ejection fraction; FS, fractional shortening; LVEDs, left ventricular end-systolic dimension; APJ, Apelin receptor (APJ).
