# Supplementary figures and images for: ELA-11 protects the heart against oxidative stress injury induced apoptosis through ERK/MAPK and PI3K/AKT signaling pathways
Source: Front Pharmacol. 2022 Sep 8;13:873614. doi: 10.3389/fphar.2022.873614 (PMC9492932; doi:10.3389/fphar.2022.873614)

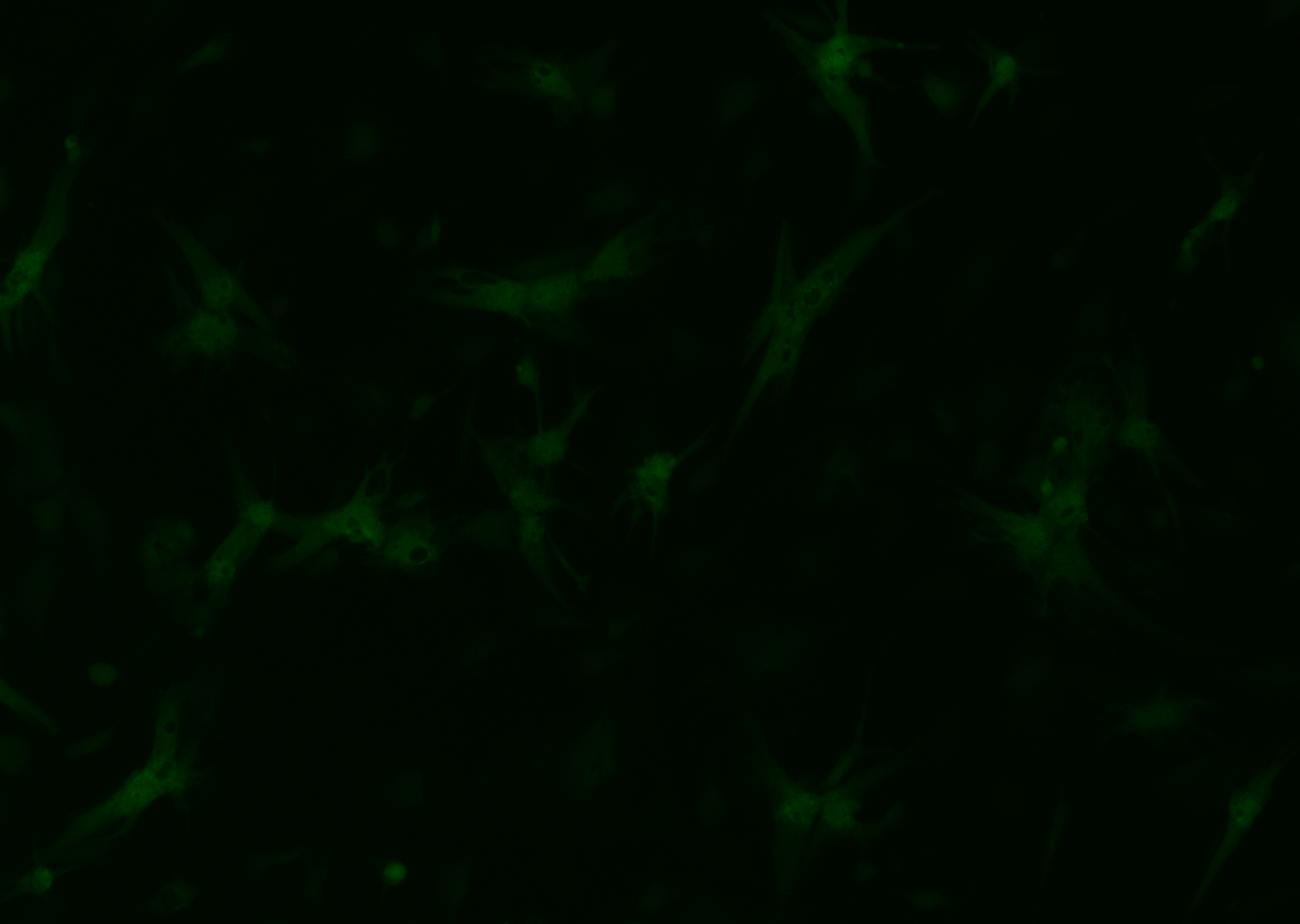

Supplement: Supplementary file 1 [file Image6.TIF]

## Slide 1
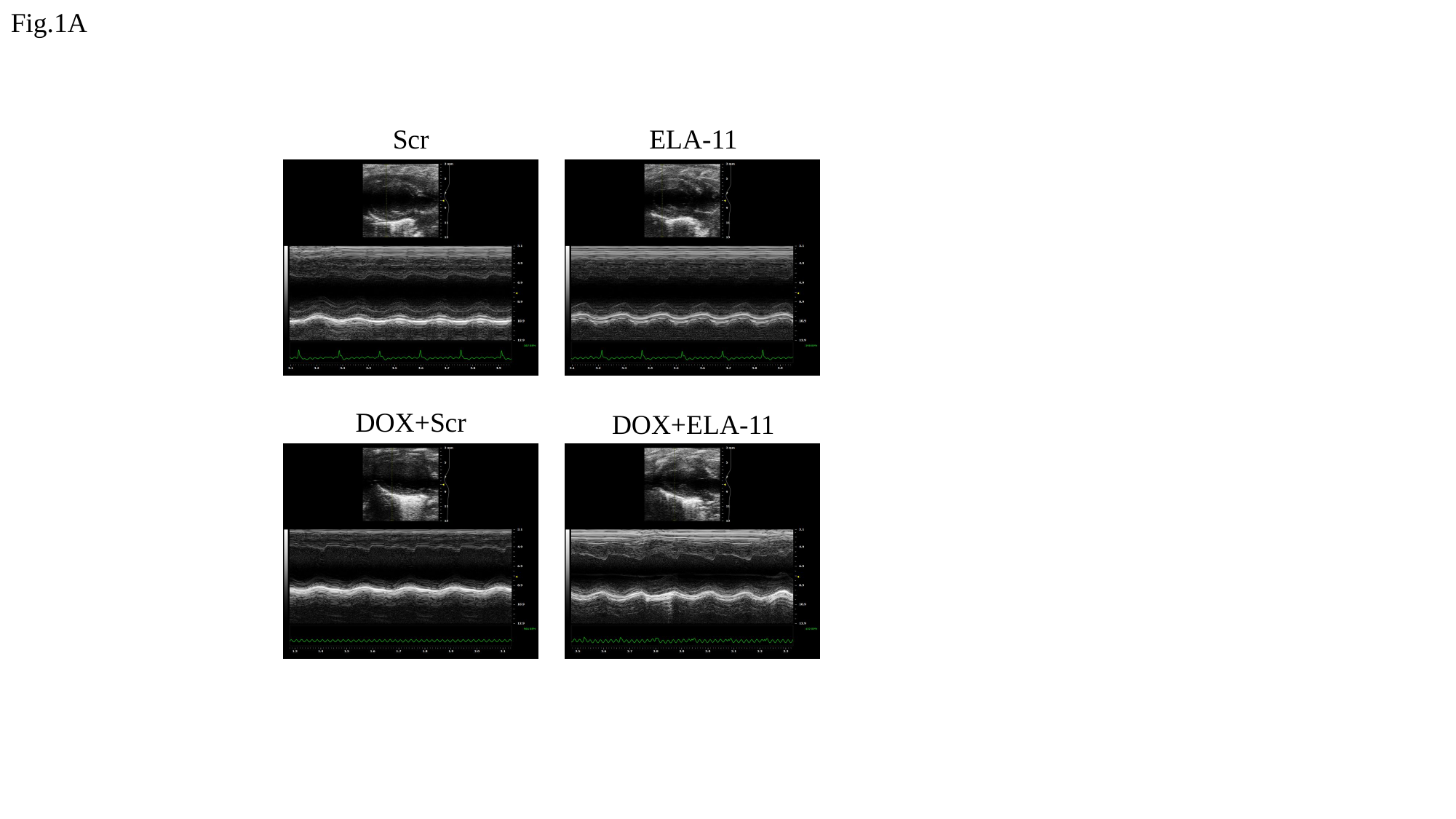

Fig.1A
Scr
ELA-11
DOX+Scr
DOX+ELA-11

## Slide 2
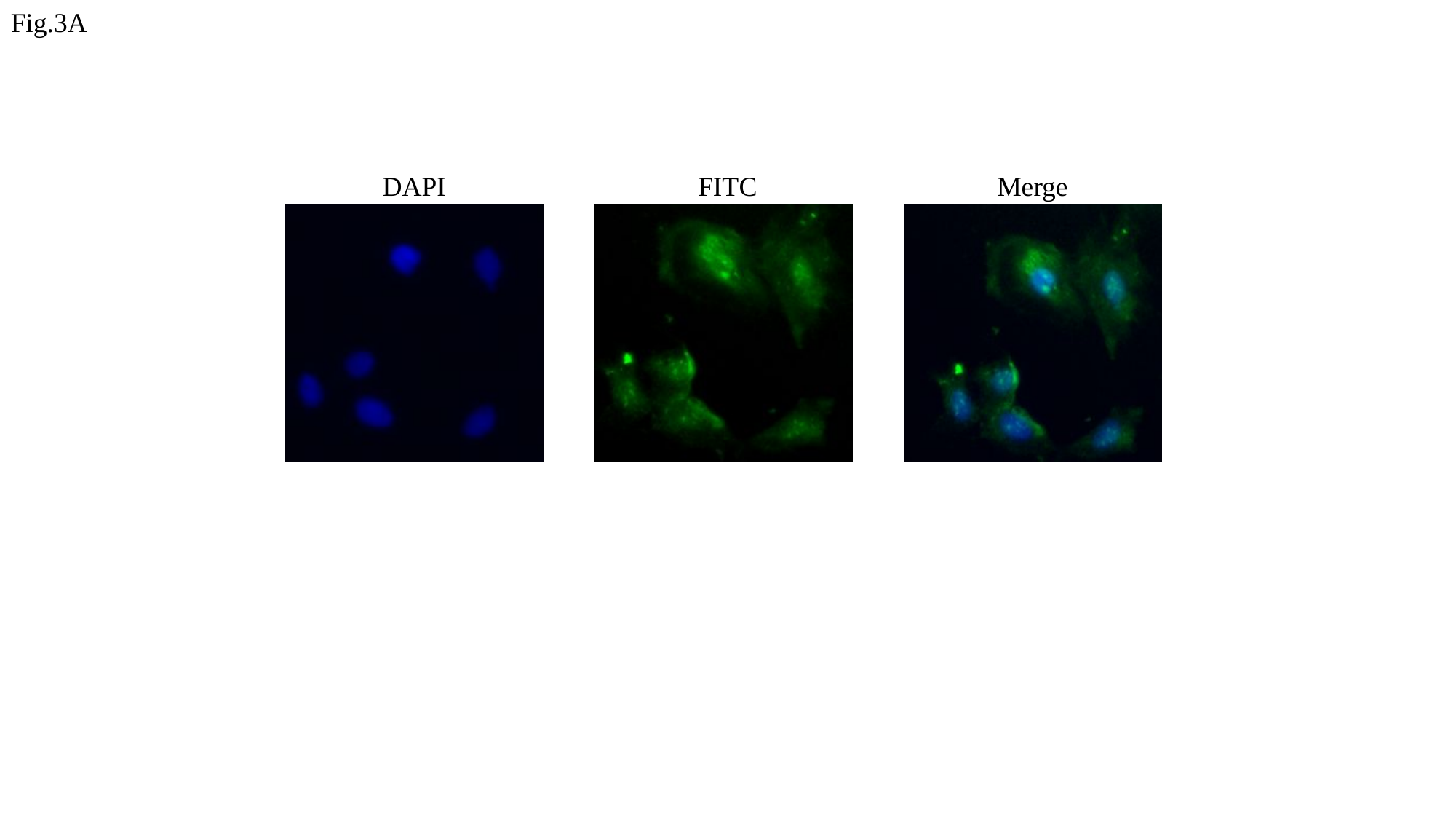

Fig.3A
DAPI
FITC
Merge

Supplement: Supplementary file 2 [file Presentation1.PPTX]

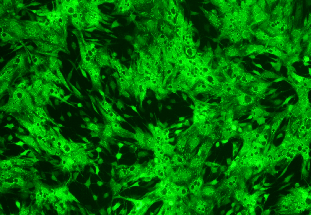

Supplement: Supplementary file 3 [file Image3.TIF]

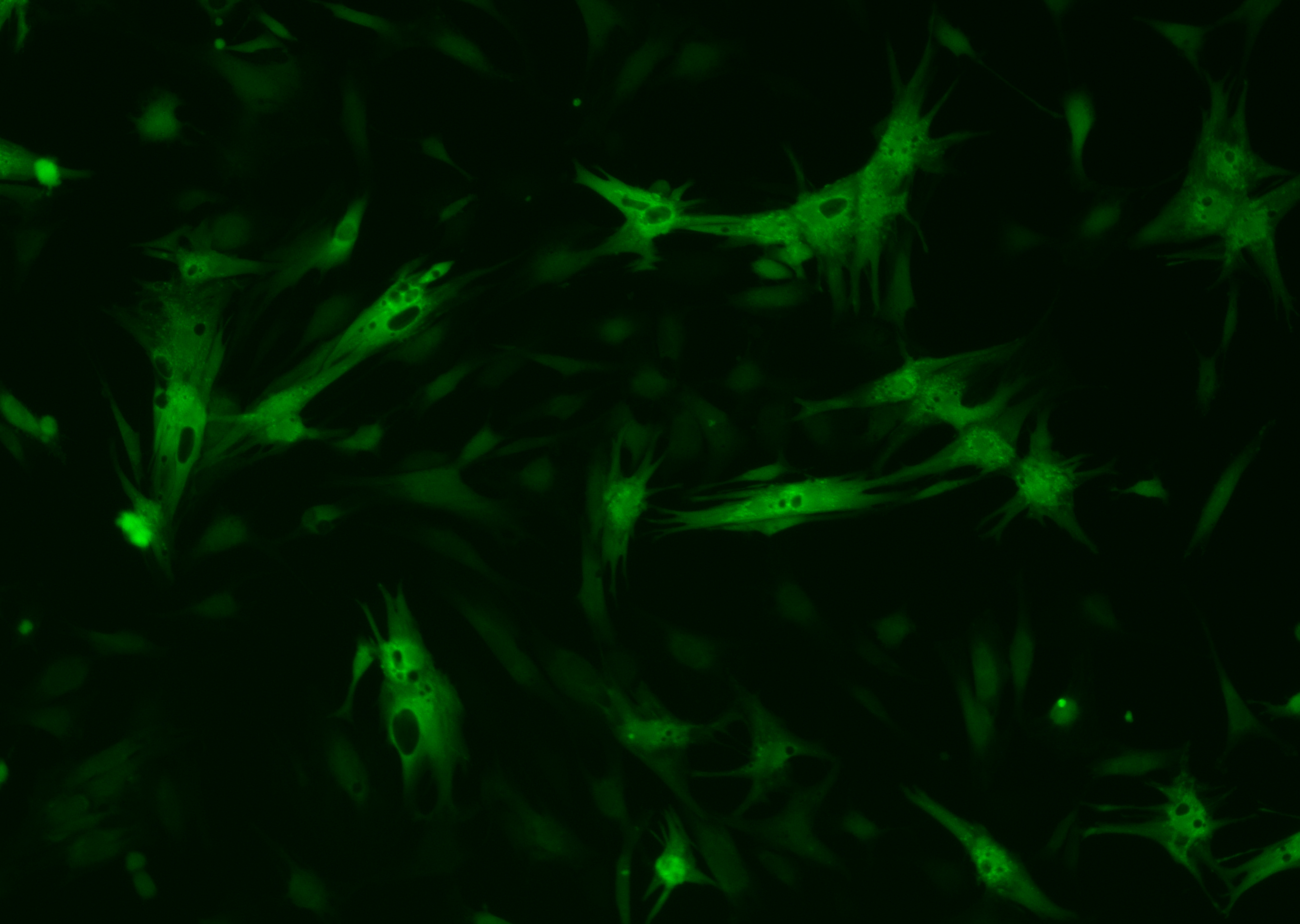

Supplement: Supplementary file 4 [file Image4.TIF]

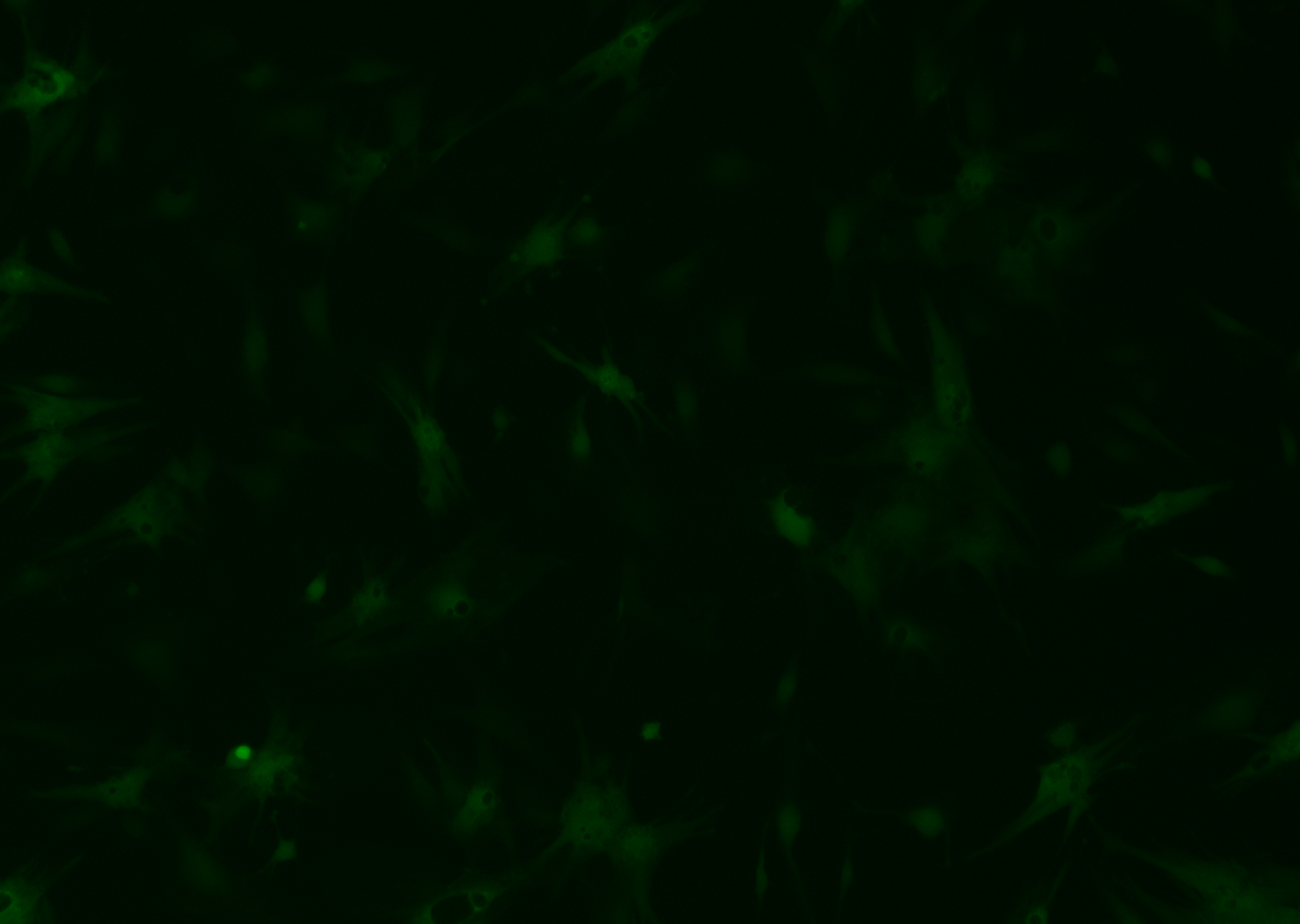

Supplement: Supplementary file 5 [file Image2.TIF]

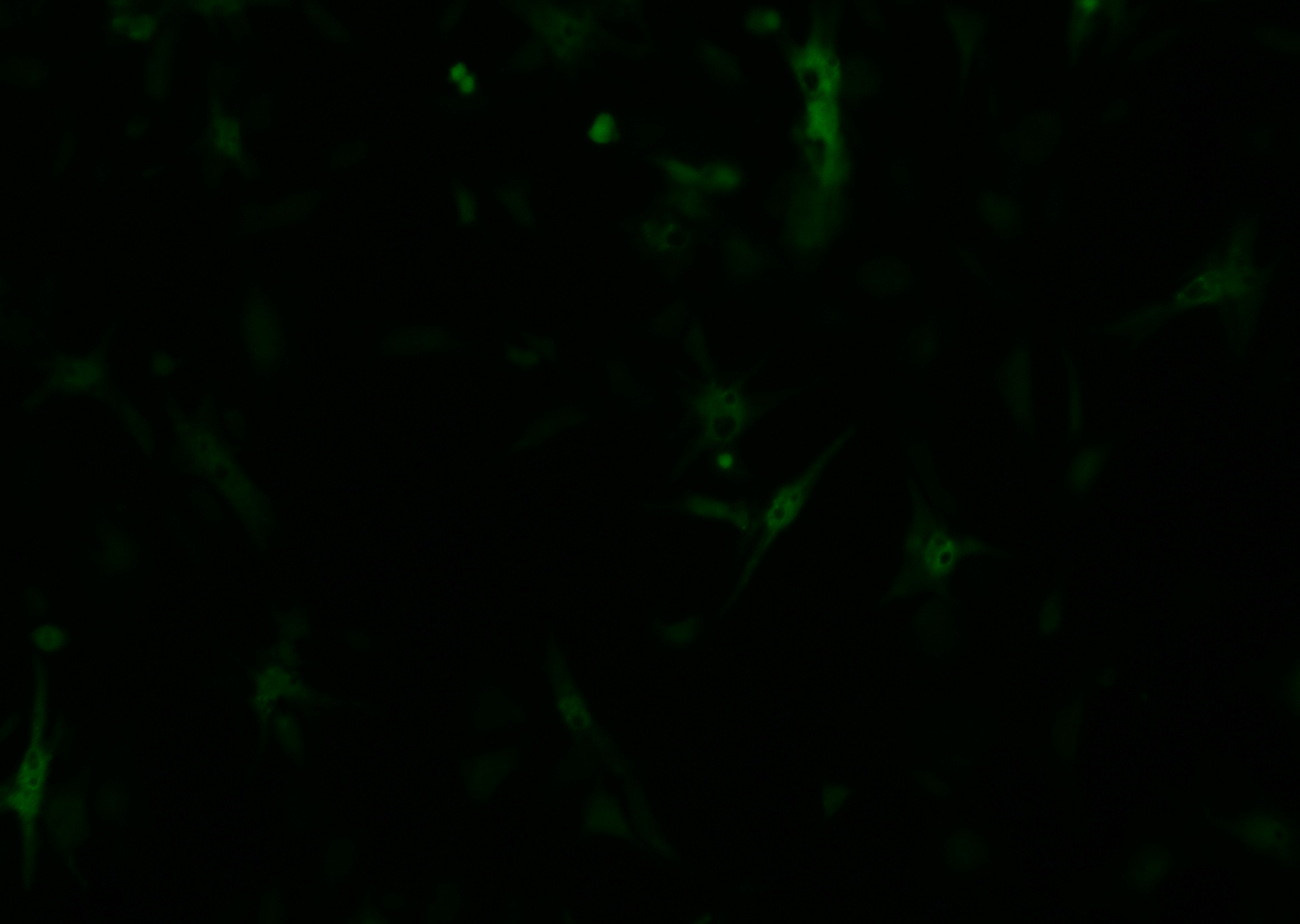

Supplement: Supplementary file 6 [file Image1.TIF]

## Slide 1
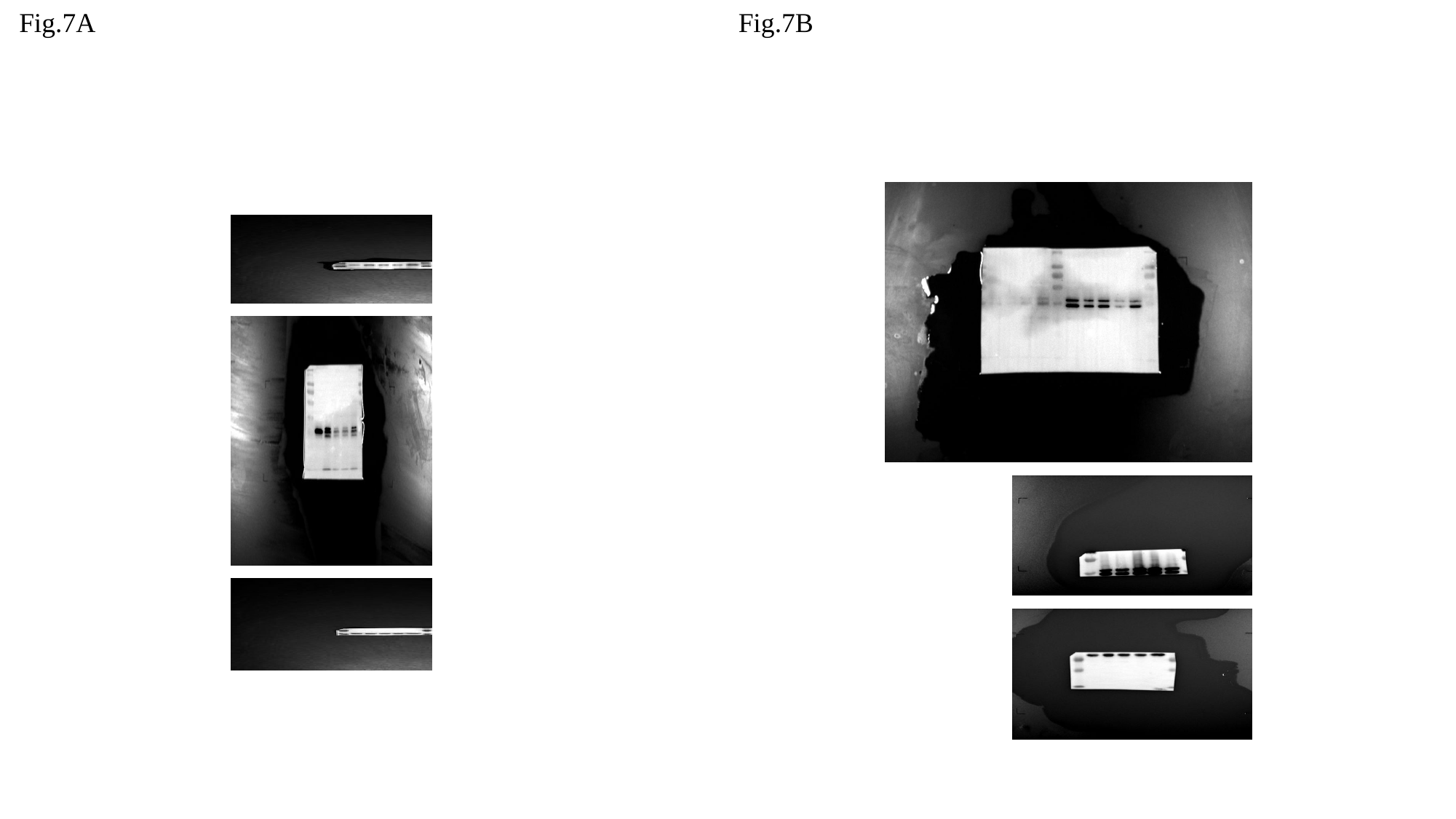

Fig.7A
Fig.7B

## Slide 2
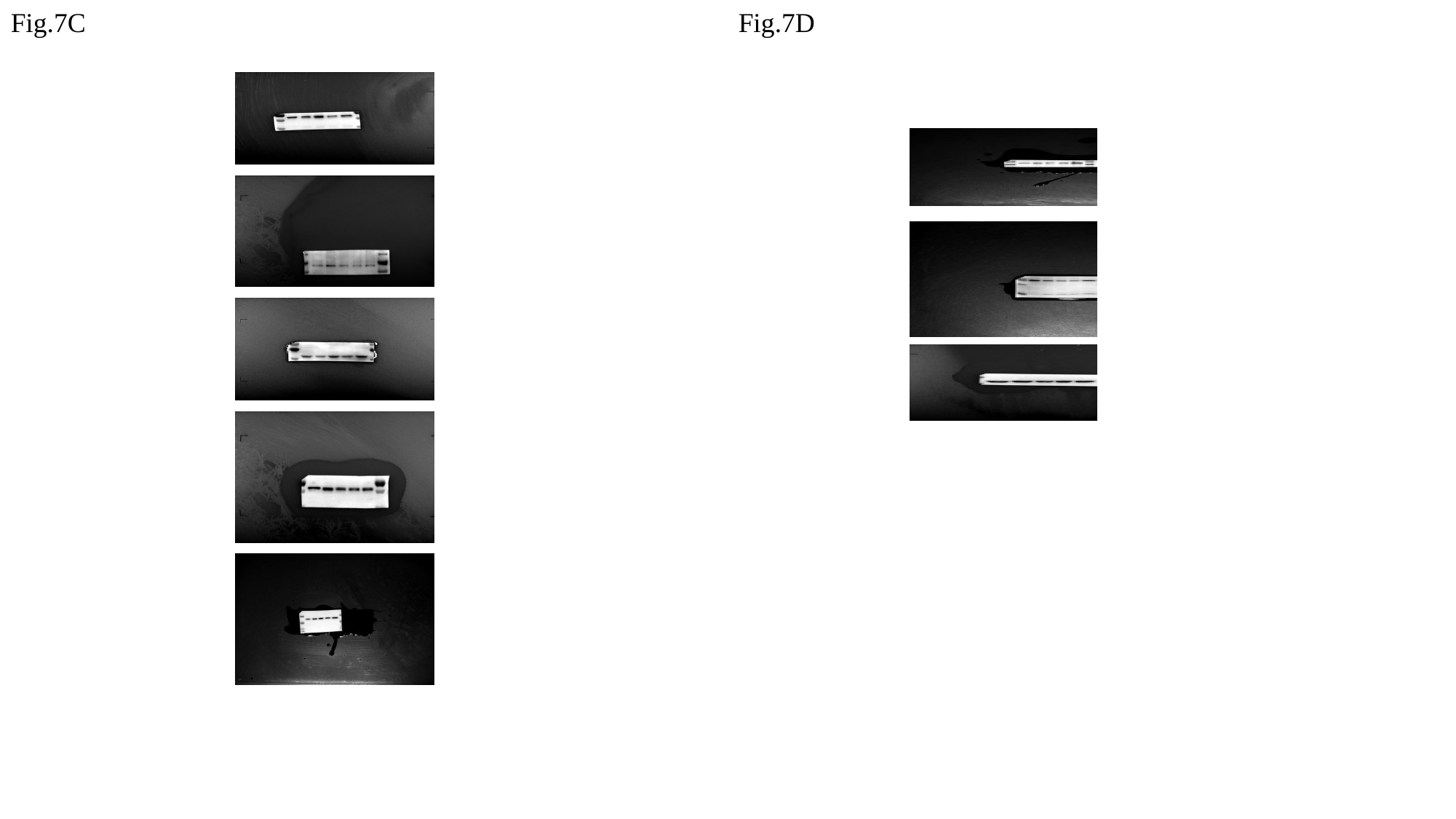

Fig.7C
Fig.7D

Supplement: Supplementary file 7 [file Presentation4.PPTX]

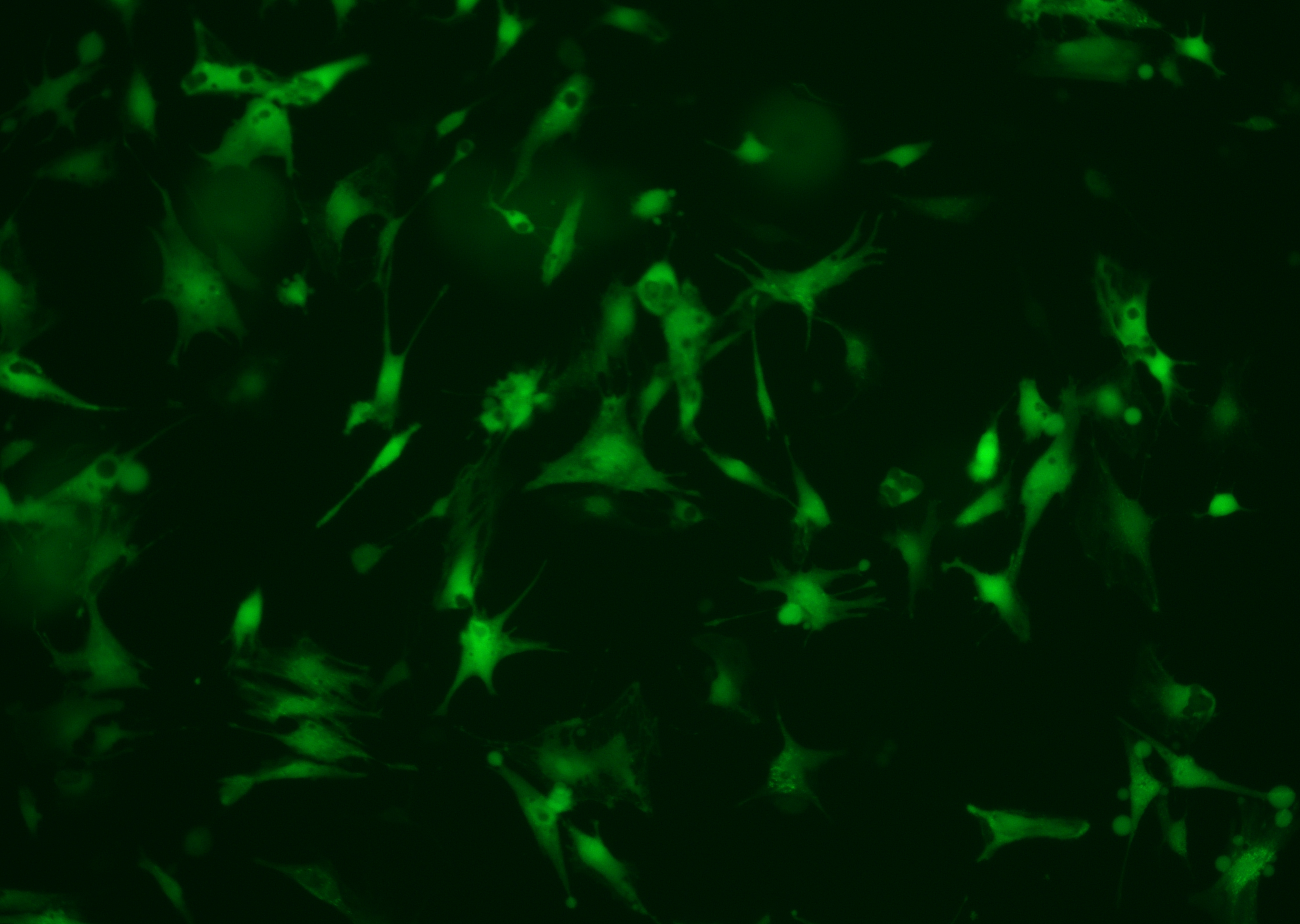

Supplement: Supplementary file 8 [file Image7.TIF]

## Slide 1
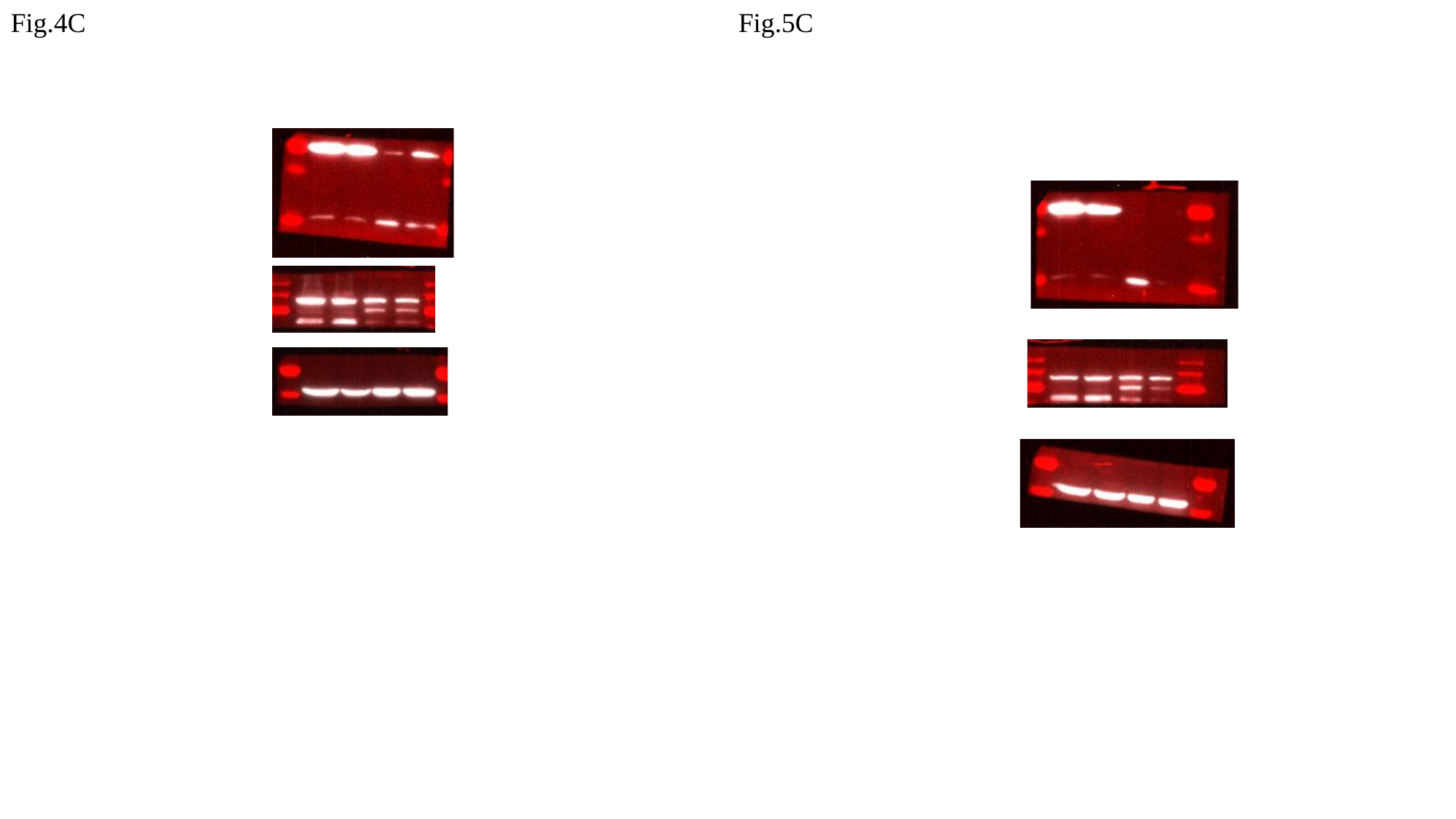

Fig.4C
Fig.5C

## Slide 2
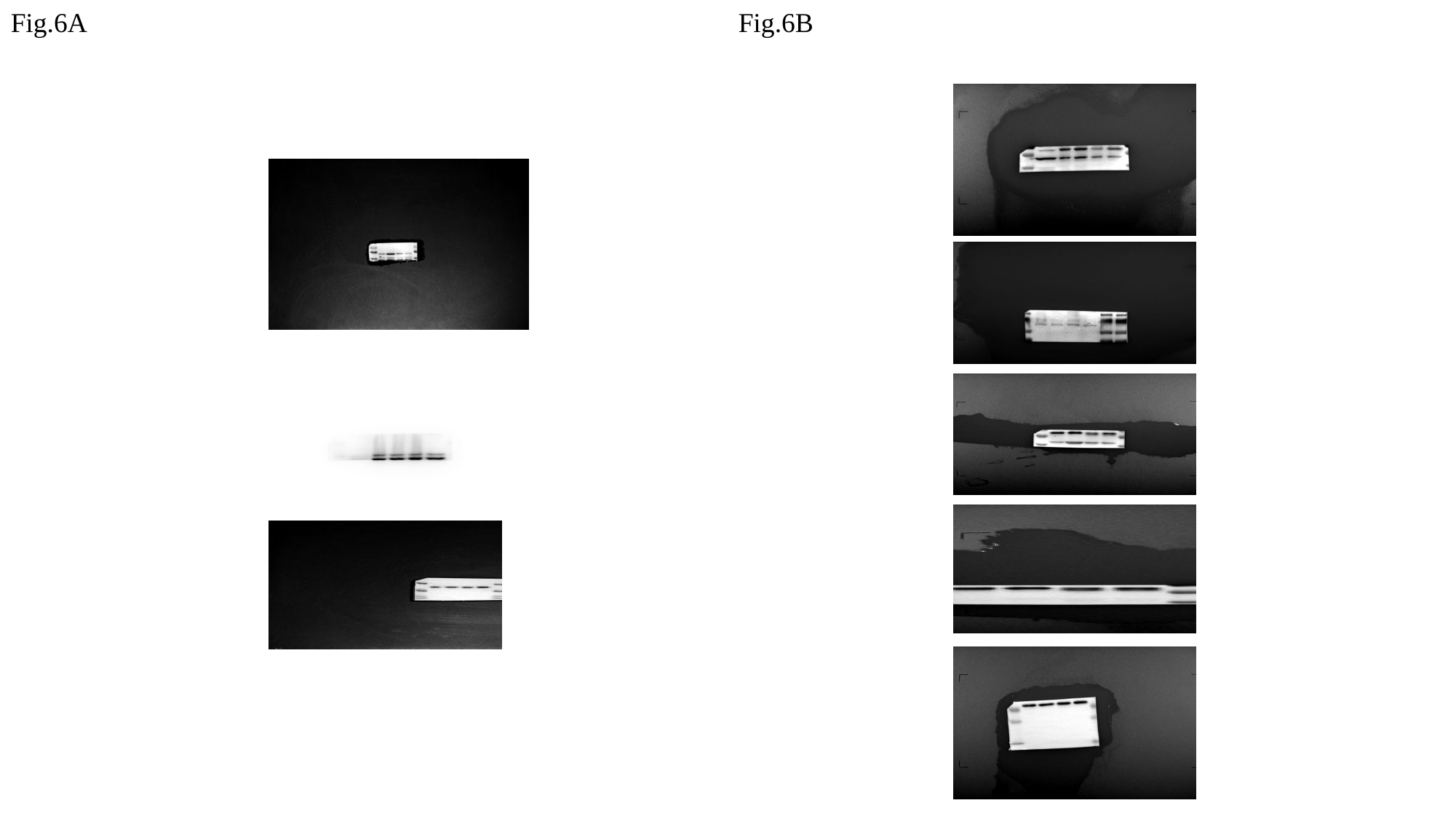

Fig.6A
Fig.6B

## Slide 3
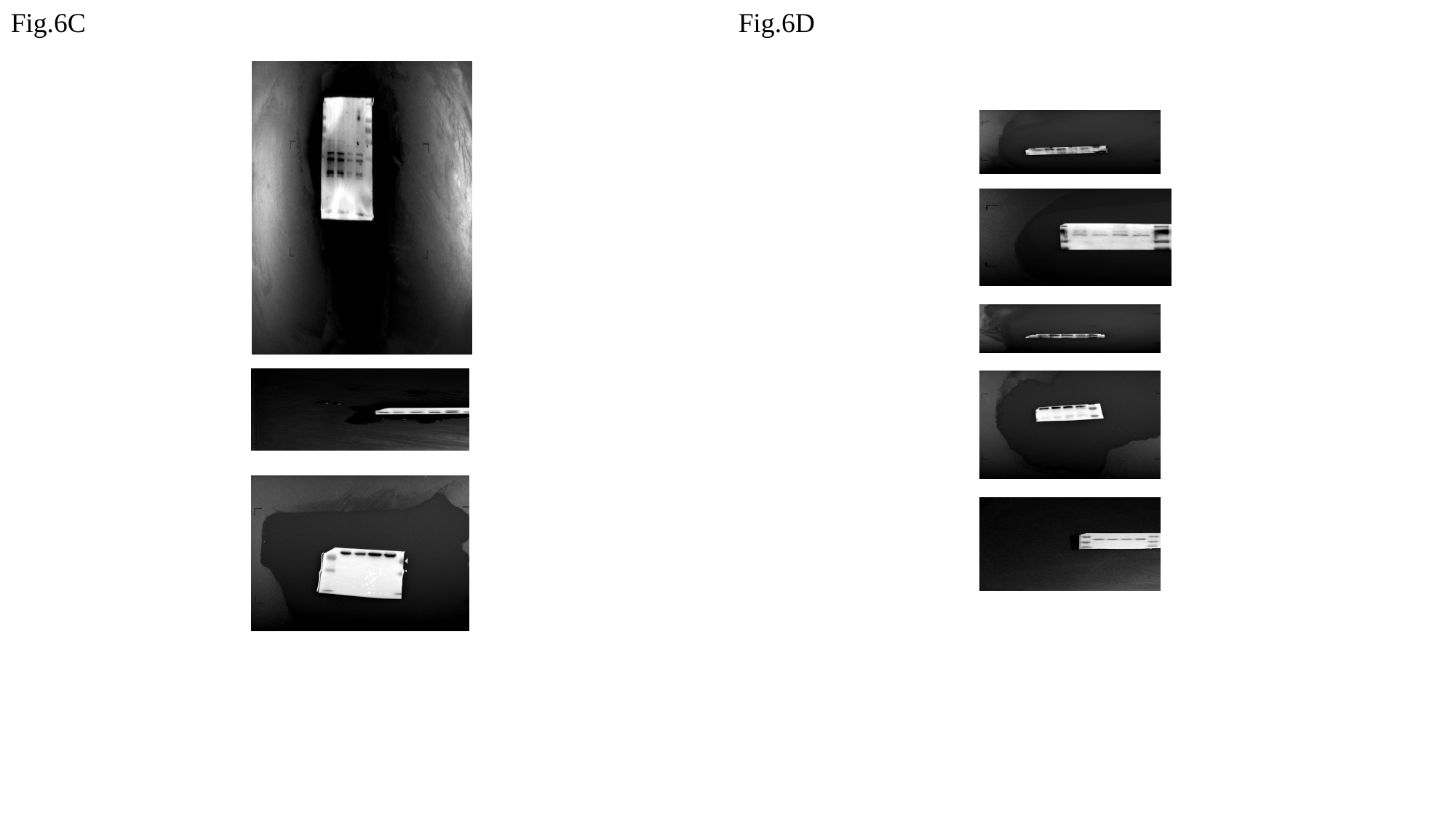

Fig.6C
Fig.6D

Supplement: Supplementary file 9 [file Presentation3.PPTX]

## Slide 1
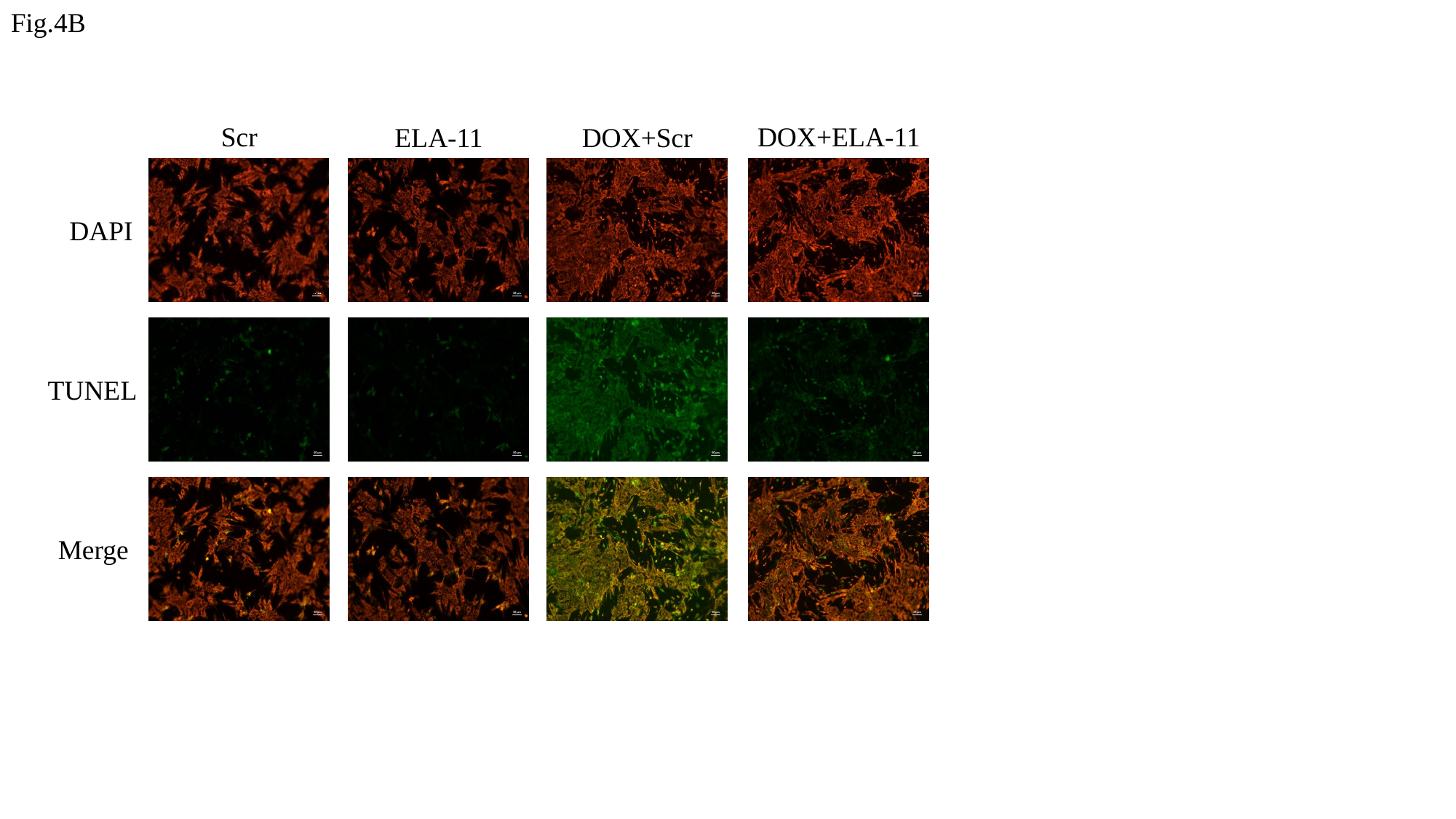

Fig.4B
Scr
DOX+ELA-11
ELA-11
DOX+Scr
DAPI
TUNEL
Merge

Supplement: Supplementary file 10 [file Presentation2.PPTX]

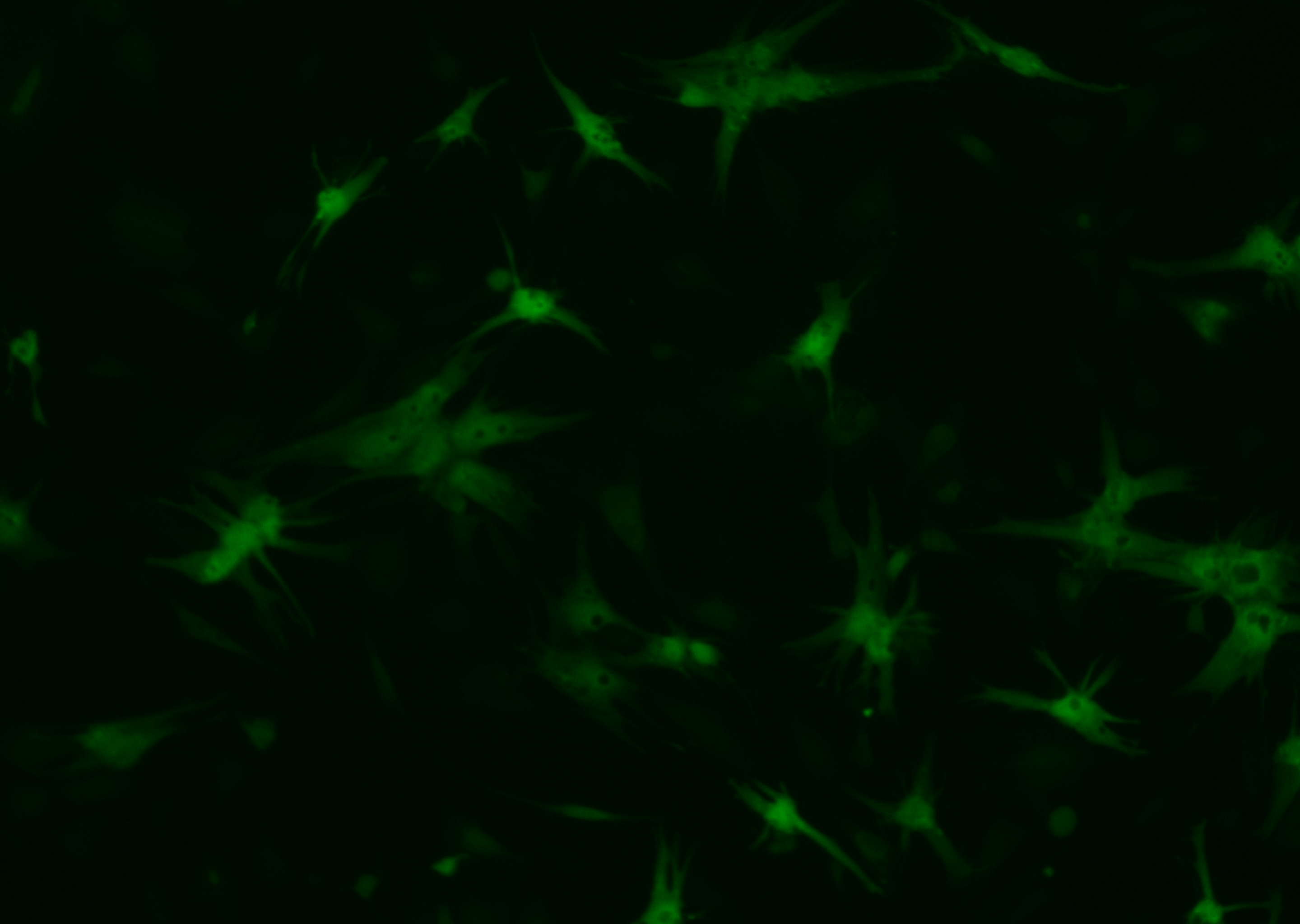

Supplement: Supplementary file 11 [file Image8.TIF]

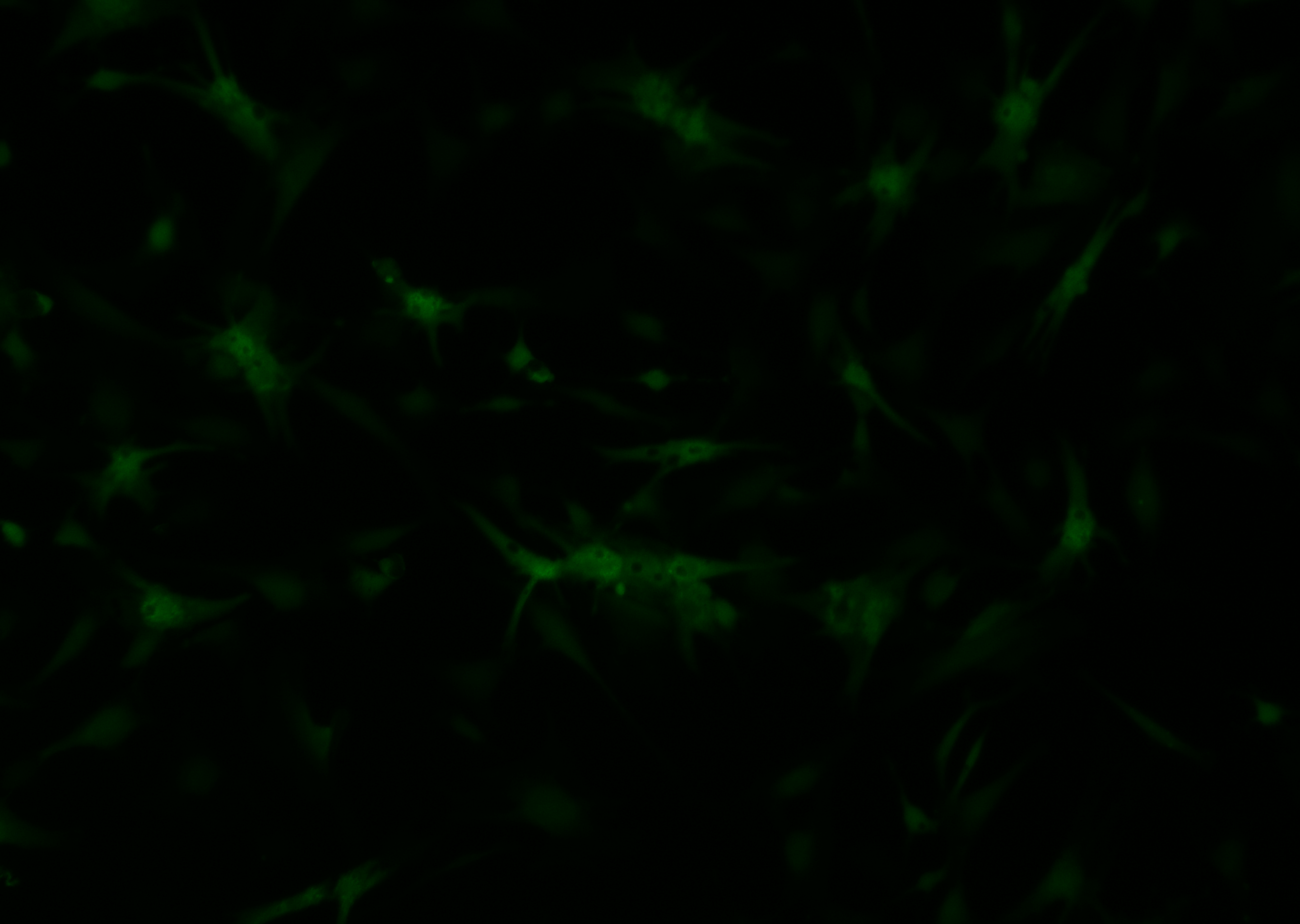

Supplement: Supplementary file 12 [file Image5.TIF]

## Slide 1
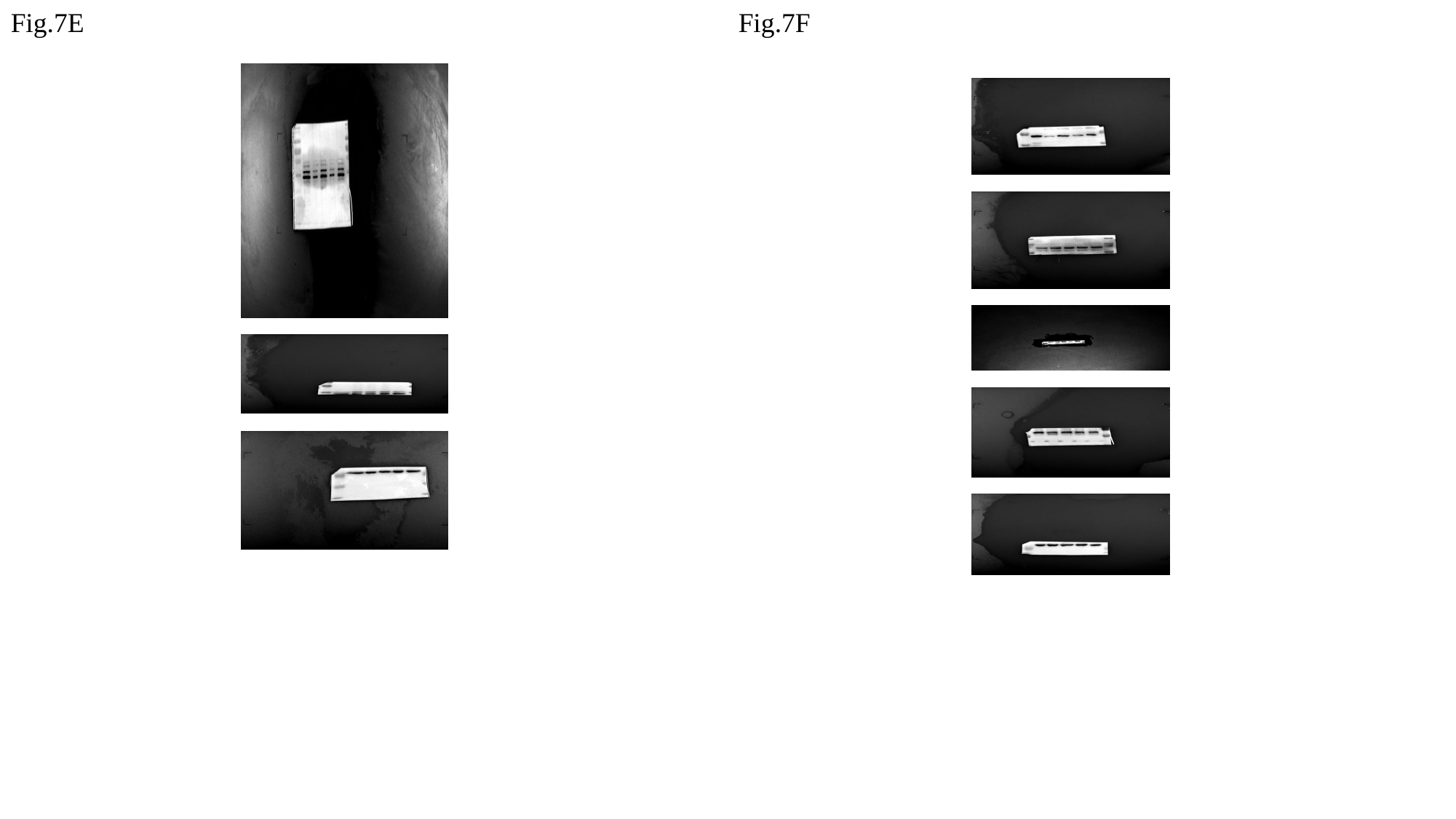

Fig.7E
Fig.7F

Supplement: Supplementary file 14 [file Presentation5.PPTX]
